# Comparison of HapMap and 1000 Genomes Reference Panels in a Large-Scale Genome-Wide Association Study

**DOI:** 10.1371/journal.pone.0167742

**Published:** 2017-01-20

**Authors:** Paul S. de Vries, Maria Sabater-Lleal, Daniel I. Chasman, Stella Trompet, Tarunveer S. Ahluwalia, Alexander Teumer, Marcus E. Kleber, Ming-Huei Chen, Jie Jin Wang, John R. Attia, Riccardo E. Marioni, Maristella Steri, Lu-Chen Weng, Rene Pool, Vera Grossmann, Jennifer A. Brody, Cristina Venturini, Toshiko Tanaka, Lynda M. Rose, Christopher Oldmeadow, Johanna Mazur, Saonli Basu, Mattias Frånberg, Qiong Yang, Symen Ligthart, Jouke J. Hottenga, Ann Rumley, Antonella Mulas, Anton J. M. de Craen, Anne Grotevendt, Kent D. Taylor, Graciela E. Delgado, Annette Kifley, Lorna M. Lopez, Tina L. Berentzen, Massimo Mangino, Stefania Bandinelli, Alanna C. Morrison, Anders Hamsten, Geoffrey Tofler, Moniek P. M. de Maat, Harmen H. M. Draisma, Gordon D. Lowe, Magdalena Zoledziewska, Naveed Sattar, Karl J. Lackner, Uwe Völker, Barbara McKnight, Jie Huang, Elizabeth G. Holliday, Mark A. McEvoy, John M. Starr, Pirro G. Hysi, Dena G. Hernandez, Weihua Guan, Fernando Rivadeneira, Wendy L. McArdle, P. Eline Slagboom, Tanja Zeller, Bruce M. Psaty, André G. Uitterlinden, Eco J. C. de Geus, David J. Stott, Harald Binder, Albert Hofman, Oscar H. Franco, Jerome I. Rotter, Luigi Ferrucci, Tim D. Spector, Ian J. Deary, Winfried März, Andreas Greinacher, Philipp S. Wild, Francesco Cucca, Dorret I. Boomsma, Hugh Watkins, Weihong Tang, Paul M. Ridker, Jan W. Jukema, Rodney J. Scott, Paul Mitchell, Torben Hansen, Christopher J. O'Donnell, Nicholas L. Smith, David P. Strachan, Abbas Dehghan

**Affiliations:** 1 Department of Epidemiology, Erasmus MC, Rotterdam, the Netherlands; 2 University of Texas Health Science Center at Houston, Houston, TX, United States of America; 3 Cardiovascular Medicine Unit, Department of Medicine, Karolinska Institutet, Stockholm, Sweden; 4 Division of Preventive Medicine, Brigham and Women's Hospital, Boston, MA, United States of America; 5 Harvard Medical School, Boston, MA, United States of America; 6 Department of Cardiology, Leiden University Medical Center, Leiden, the Netherlands; 7 Department of Gerontology and Geriatrics, Leiden University Medical Center, Leiden, the Netherlands; 8 Steno Diabetes Center Copenhagen, Gentofte, Denmark; 9 Novo Nordisk Foundation Center For Basic Metabolic Research, Section of Metabolic Genetics, Faculty of Health and Medical Sciences, University of Copenhagen, Copenhagen, Denmark; 10 Institute for Community Medicine, University Medicine Greifswald, Greifswald, Germany; 11 Vth Department of Medicine, Medical Faculty Mannheim, Heidelberg University, Mannheim, Germany; 12 Department of Neurology, Boston University School of Medicine, Boston, MA, United States of America; 13 Framingham Heart Study, Population Sciences Branch, Division of Intramural Research National Heart Lung and Blood Institute, National Institutes of Health, Framingham, MA, United States of America; 14 Centre for Vision Research, Department of Ophthalmology, and Westmead Institute for Medical Research, University of Sydney, Sydney, Australia; 15 Public Health Stream, Hunter Medical Research Institute, University of Newcastle, Newcastle, Australia; 16 School of Medicine and Public Health, University of Newcastle, Newcastle, Australia; 17 Centre for Cognitive Ageing and Cognitive Epidemiology, University of Edinburgh, Edinburgh, United Kingdom; 18 Centre for Genomic and Experimental Medicine, University of Edinburgh, Edinburgh, United Kingdom; 19 Queensland Brain Institute, University of Queensland, Brisbane, Australia; 20 Istituto di Ricerca Genetica e Biomedica, Consiglio Nazionale delle Ricerche, Monserrato, Cagliari, Italy; 21 Division of Epidemiology and Community Health, University of Minnesota, Minneapolis, MN, United States of America; 22 Department of Biological Psychology, Netherlands Twin Register, VU University, Amsterdam, the Netherlands; 23 EMGO+ institute, VU University & VU medical center, Amsterdam, the Netherlands; 24 Center for Thrombosis and Hemostasis (CTH), University Medical Center of the Johannes Gutenberg University Mainz, Mainz, Germany; 25 Department of Medicine, University of Washington, Seattle WA, United States of America; 26 Division of Infection and Immunology, UCL, London, United Kingdom; 27 Department of Twin Research and Genetic Epidemiology, Kings College London, London, United Kingdom; 28 Translational Gerontology Branch, National Institute on Aging, Baltimore, MD, United States of America; 29 Institute of Medical Biostatistics, Epidemiology and Informatics, University Medical Center of the Johannes Gutenberg University Mainz, Mainz, Germany; 30 Division of Biostatistics, University of Minnesota, Minneapolis, MN, United States of America; 31 Department of Numerical Analysis and Computer Science, Stockholm University, Stockholm, Sweden; 32 Department of Biostatistics, Boston University School of Public Health, Boston, MA, United States of America; 33 Institute of Cardiovascular and Medical Sciences, University of Glasgow, Glasgow, United Kingdom; 34 Institute of Clinical Chemistry and Laboratory Medicine, University Medicine Greifswald, Greifswald, Germany; 35 Institute for Translational Genomics and Population Sciences, Los Angeles Biomedical Research Institute at Harbor/UCLA Medical Center, Torrance, CA, United States of America; 36 Division of Genomic Outcomes, Department of Pediatrics, Harbor-UCLA Medical Center, Torrance, CA, United States of America; 37 Royal College of Surgeons in Ireland, Department of Psychiatry, Education and Research Centre, Beaumont Hospital, Dublin, Ireland; 38 University College Dublin, UCD Conway Institute, Centre for Proteome Research, UCD, Belfield, Dublin, Ireland; 39 Institute of Preventive Medicine, Bispebjerg and Frederiksberg Hospital, The Capital Region, Copenhagen, Denmark; 40 NIHR Biomedical Research Centre at Guy’s and St. Thomas’ Foundation Trust, London, United Kingdom; 41 Geriatric Unit, Azienda Sanitaria Firenze (ASF), Florence, Italy; 42 Royal North Shore Hospital, Sydney University, Sydney, Australia; 43 Department of Hematology, Erasmus MC, Rotterdam, the Netherlands; 44 Neuroscience Campus Amsterdam, Amsterdam, the Netherlands; 45 Institute of Cardiovascular and Medical Sciences, University of Glasgow, Glasgow, United Kingdom; 46 BHF Glasgow Cardiovascular Research Centre, Faculty of Medicine, Glasgow, United Kingdom; 47 Institute of Clinical Chemistry and Laboratory Medicine, University Medical Center, Johannes Gutenberg University Mainz, Mainz, Germany; 48 Interfaculty Institute for Genetics and Functional Genomics, University Medicine Greifswald, Greifswald, Germany; 49 Department of Biostatistics, University of Washington, Seattle, WA, United States of America; 50 Department of Human Genetics, Wellcome Trust Sanger Institute, Hinxton, Cambridge, United Kingdom; 51 Public Health Stream, Hunter Medical Research Institute, and School of Medicine and Public Health, University of Newcastle, Newcastle, Australia; 52 Alzheimer Scotland Dementia Research Centre, University of Edinburgh, Edinburgh, United Kingdom; 53 Laboratory of Neurogenetics, National Institute on Aging, Bethesda, MD, United States of America; 54 Department of Internal Medicine, Erasmus MC, Rotterdam, the Netherlands; 55 School of Social and Community Medicine, University of Bristol, Bristol, United Kingdom; 56 Department of Molecular Epidemiology, Leiden University Medical Center, Leiden, the Netherlands; 57 Department of General and Interventional Cardiology, University Heart Centre, University Medical Center Hamburg-Eppendorf, Hamburg, Germany; 58 German Center for Cardiovascular Research (DZHK), Partner Site Hamburg, Lübeck, Kiel, Hamburg, Germany; 59 Department of Medicine, Epidemiology, and Health Services, University of Washington, Seattle WA, United States of America; 60 Group Health Research Institute, Group Health Cooperative, Seattle WA, United States of America; 61 Institute of Cardiovascular and Medical Sciences, Faculty of Medicine, University of Glasgow, Glasgow, United Kingdom; 62 Institute of Medical Biostatistics, Epidemiology and Informatics, University Medical Center of the Johannes Gutenberg University Mainz, Mainz, Germany; 63 Department of Epidemiology, Harvard T.H. Chan School of Public Health, Boston, MS, United States of America; 64 Los Angeles BioMedical Research Institute at Harbor-UCLA Medical Center, Institute for Translational Genomics and Population Sciences, Torrance, CA, United States of America; 65 Division of Genomic Outcomes, Departments of Pediatrics & Medicine, Harbor-UCLA Medical Center, Torrance, CA, United States of America; 66 Department of Psychology, University of Edinburgh, Edinburgh, United Kingdom; 67 Synlab Academy, Synlab Holding Deutschland GmbH, Mannheim, Germany; 68 Clinical Institute of Medical and Chemical Laboratory Diagnostics, Medical University of Graz, Graz, Austria; 69 Institute for Immunology and Transfusion Medicine, University Medicine Greifswald, Greifswald, Germany; 70 Preventive Cardiology and Preventive Medicine, Center for Cardiology, University Medical Center of the Johannes Gutenberg-University Mainz, Mainz, Germany; 71 Center for Thrombosis and Hemostasis (CTH), University Medical Center of the Johannes Gutenberg-University Mainz, Mainz, Germany; 72 German Center for Cardiovascular Research (DZHK), Partner Site RhineMain, Mainz, Germany; 73 Cardiovascular Medicine Dept/Radcliffe Dept of Medicine, Wellcome Trust Centre for Human Genetics, University of Oxford, Oxford, United Kingdom; 74 Durrer Center for Cardiogenetic Research, Amsterdam, the Netherlands; 75 Interuniversity Cardiology Institute of the Netherlands, Utrecht, the Netherlands; 76 Information based Medicine Program, Hunter Medical Research Institute, University of Newcastle, Newcastle, Australia; 77 School of Biomedical Sciences and Pharmacy, University of Newcastle, Newcastle, Australia; 78 Novo Nordisk Foundation Centre for Basic Metabolic Research, Section of Metabolic Genetics, Faculty of Health and Medical Sciences, University of Copenhagen, Copenhagen, Denmark; 79 Cardiology Division, Massachusetts General Hospital, Boston, MA, United States of America; 80 Department of Epidemiology, University of Washington, Seattle WA, United States of America; 81 Seattle Epidemiologic Research and Information Center, Department of Veteran Affairs Office of Research and Development, Seattle, WA, United States of America; 82 Population Health Research Institute, St George's, University of London, London, United Kingdom; 83 Department of Epidemiology and Biostatistics, Imperial College London, London, United Kingdom; Kunming Institute of Zoology, Chinese Academy of Sciences, CHINA

## Abstract

An increasing number of genome-wide association (GWA) studies are now using the higher resolution 1000 Genomes Project reference panel (1000G) for imputation, with the expectation that 1000G imputation will lead to the discovery of additional associated loci when compared to HapMap imputation. In order to assess the improvement of 1000G over HapMap imputation in identifying associated loci, we compared the results of GWA studies of circulating fibrinogen based on the two reference panels. Using both HapMap and 1000G imputation we performed a meta-analysis of 22 studies comprising the same 91,953 individuals. We identified six additional signals using 1000G imputation, while 29 loci were associated using both HapMap and 1000G imputation. One locus identified using HapMap imputation was not significant using 1000G imputation. The genome-wide significance threshold of 5×10^−8^ is based on the number of independent statistical tests using HapMap imputation, and 1000G imputation may lead to further independent tests that should be corrected for. When using a stricter Bonferroni correction for the 1000G GWA study (*P*-value < 2.5×10^−8^), the number of loci significant only using HapMap imputation increased to 4 while the number of loci significant only using 1000G decreased to 5. In conclusion, 1000G imputation enabled the identification of 20% more loci than HapMap imputation, although the advantage of 1000G imputation became less clear when a stricter Bonferroni correction was used. More generally, our results provide insights that are applicable to the implementation of other dense reference panels that are under development.

## Introduction

Most genome-wide association (GWA) studies to date have used their genotyped single nucleotide polymorphisms (SNPs) to impute about 2.5 million SNPs detected in the Phase 2 version of the HapMap Project (HapMap) [1–13], including mostly common SNPs with a minor allele frequency (MAF) of over 5%. HapMap imputation enabled the interrogation of most common SNPs possible, even while meta-analyzing studies that used different genotyping arrays with low overlap [1]. However, low-frequency and rare variants are not well covered in the HapMap panel [14]. In addition, genetic variants other than SNPs, such as small insertion/deletions (indels) and large structural variants, are not included in HapMap-based imputed projects, and may be possible sources of missing explained heritability.

In contrast, the more recently released Phase 1 version 3 of the 1000 Genomes Project (1000G) is based on a larger set of individuals [15], and comprises nearly 40 million variants, including 1.4 million indels. 1000G allows the interrogation of most common and low-frequency variants (MAF > 1%), and rare variants (MAF < 1%) that were previously not covered [16]. In general, improving reference panels can lead to the identification of additional significant loci both through the addition of new variants and the improved imputation of known variants. 1000G imputation may thus have several advantages, but given that the denser 1000G imputation comes at the cost of an increased computational and analytical burden, it is important to estimate the observed benefits in practice. Furthermore, such empirical data is needed to make informed decisions in the future on the use of newer reference panels such as UK10K, and the Haplotype Reference Consortium [17, 18]. While several GWA studies using 1000G imputation have been published or are in progress, their sample size differs from the previous GWA studies using HapMap imputation, making comparison difficult. Therefore, with the aim of evaluating the benefits of using 1000G imputation in GWA studies compared to HapMap imputation, we carried out meta-analyses of GWA studies of circulating fibrinogen concentration (a quantitative trait), using both HapMap and 1000G imputed data on the same set of 91,953 individuals.

## Results

**Fig 1:**
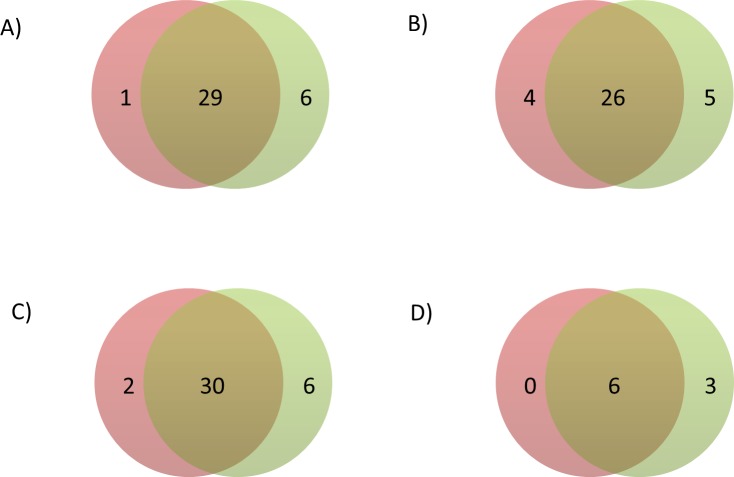
Venn diagram of the number of loci significant using HapMap (left circle) and 1000G (right circle) imputation in A) the main analysis, B) the sensitivity analysis applying a significance threshold of 2.5×10^−8^ to the 1000G GWA analysis, C) the sensitivity analysis without using genomic control corrections, and D) the sensitivity analysis excluding studies that used different imputation software, analysis software, or covariates in the HapMap and 1000G GWA analyses.

Baseline characteristics of the participants for each of the included studies are shown in S1 Table, and genomic inflation factors are shown separately for the HapMap and 1000G GWA studies in S2 Table. The HapMap GWA study included 2,749,429 SNPs, and the 1000G GWA study included 10,883,314 variants. Summary statistics for all variants in the HapMap and 1000G GWA studies are available via the dbGAP CHARGE Summary Results site [19]. Using a genome-wide significance threshold of 5×10^−8^, a total of 1,210 SNPs across 30 loci were associated with circulating fibrinogen concentration in the HapMap imputed GWA study compared with 4,096 variants across 35 loci in the 1000G imputed GWA study (S1 Fig and S2 Fig). These loci are described in further detail in S3 Table. Of these loci, six were associated only in the 1000G GWA study and one was associated only in the HapMap GWA study, while 29 were overlapping (Fig 1A). The HapMap and 1000G lead variants of non-overlapping loci are described in Table 1, and leads variants of overlapping loci are described in Table 2. Among significant loci, the correlation coefficient across cohorts of the beta coefficients, *P*-values, and imputation quality scores of HapMap and 1000G lead variants were 0.925, 0.998, and 0.435 respectively (S3 Fig).

**Table 1:** Non-overlapping loci that were significant in either the HapMap or 1000G GWA studies.

	HapMap					1000G				
Locus	Lead Variant	Beta	*P*-value	MAF	Imputation Quality	Lead Variant	Beta	*P*-value	MAF	Imputation Quality
*Significant in 1000G*										
1q42.13	rs10489615	0.0052	8.3×10^−07^	0.38	0.97	rs10864726	0.0059	1.1×10^−08^	0.40	0.96
3q21.1	rs16834024	0.0173	1.4×10^−07^	0.03	0.79	rs1976714	0.0064	7.5×10^−09^	0.35	0.89
4p16.3	rs2699429	0.0060	1.3×10^−07^	0.43	0.87	rs59950280	0.0080	2.5×10^−11^	0.34	0.80
7p15.3	rs1029738	0.0057	3.2×10^−07^	0.30	1.00	rs61542988	0.0065	3.1×10^−08^	0.25	0.98
8p23.1	rs7004769	0.0062	1.4×10^−06^	0.20	1.00	rs7012814	0.0061	8.0×10^−09^	0.47	0.91
11q12.2	rs7935829	0.0056	5.6×10^−08^	0.40	0.99	rs11230201	0.0060	3.0×10^−09^	0.41	0.99
*Significant in HapMap*										
6p21.3	rs12528797	0.0095	8.5×10^−09^	0.11	0.98	rs116134220	0.0082	7.9×10^−06^	0.49	0.89

Further detail about these loci and the lead variants is provided in S3 Table. *Abbreviations*: HapMap refers to the GWA study using imputation based on the HapMap project. 1000G refers to the GWA study using imputation based on the 1000 Genomes Project. Variants were coded according to the fibrinogen increasing allele. MAF refers to minor allele frequency.

**Table 2:** Overlapping loci that were significant in both the HapMap and 1000G GWA studies.

	HapMap					1000G				
Locus	Lead Variant	Beta	*P*-value	MAF	Imputation Quality	Lead Variant	Beta	*P*-value	MAF	Imputation Quality
1p31.3	rs4655582	0.0069	4.8×10^−11^	0.38	0.98	rs2376015	0.0075	5.1×10^−12^	0.35	0.91
1q21.3	rs8192284	0.0115	8.9×10^−29^	0.40	0.97	rs61812598	0.0114	1.8×10^−28^	0.39	0.99
1q44	rs12239046	0.0103	9.7×10^−21^	0.38	0.99	rs12239046	0.0102	9.8×10^−22^	0.38	0.99
2q12	rs1558643	0.0066	5.8×10^−10^	0.40	0.99	rs1558643	0.0063	6.0×10^−10^	0.40	0.98
2q13	rs6734238	0.0106	1.7×10^−23^	0.41	0.99	rs6734238	0.0106	3.7×10^−24^	0.41	1.00
2q34	rs715	0.0092	9.1×10^−14^	0.32	0.92	rs715	0.0082	1.7×10^−13^	0.32	0.89
2q37.3	rs1476698	0.0075	4.2×10^−12^	0.36	1.00	rs59104589	0.0081	2.4×10^−14^	0.34	0.98
3q22.2	rs548288	0.0113	6.6×10^−21^	0.24	0.99	rs150213942	0.0117	3.1×10^−21^	0.23	0.95
4q31.3	rs2227401	0.0311	4.7×10^−134^	0.21	0.95	rs72681211	0.0313	1.3×10^−142^	0.20	0.99
5q31.1	rs1012793	0.0208	4.4×10^−60^	0.21	0.98	rs1012793	0.0207	1.0×10^−58^	0.20	0.98
7p21.1	rs10950690	0.0071	9.9×10^−12^	0.48	0.94	rs12699921	0.0071	1.3×10^−12^	0.47	0.98
7q14.2	rs2710804	0.0061	9.3×10^−09^	0.38	0.98	rs2710804	0.0057	4.3×10^−08^	0.38	0.99
7q36.1	rs13226190	0.008	2.2×10^−10^	0.21	0.99	rs13234724	0.0076	1.6×10^−09^	0.21	0.99
8q24.3	rs7464572	0.0066	2.4×10^−09^	0.40	0.98	rs11136252	0.0056	4.6×10^−08^	0.42	0.96
9q22.2	rs7873907	0.006	5.4×10^−09^	0.50	0.96	rs3138493	0.006	3.5×10^−09^	0.48	0.98
10q21.3	rs10761756	0.0093	5.4×10^−20^	0.48	1.00	rs7916868	0.0097	1.2×10^−21^	0.49	0.97
11p12	rs7937127	0.0083	2.3×10^−10^	0.18	0.99	rs7934094	0.0081	2.9×10^−10^	0.22	0.90
12q13.12	rs1521516	0.0072	3.0×10^−11^	0.36	1.00	12:51042486	0.0073	4.9×10^−12^	0.36	0.98
12q24.12	rs3184504	0.0066	1.1×10^−10^	0.49	0.97	rs4766897	0.009	3.8×10^−12^	0.34	0.64
14q24.1	rs194741	0.0092	8.3×10^−14^	0.25	0.95	rs194714	0.0086	3.7×10^−13^	0.25	0.97
15q15.1	rs1703755	0.0088	1.8×10^−09^	0.14	0.96	rs8026198	0.009	5.9×10^−10^	0.15	0.93
15q21.2	rs12915052	0.0069	2.4×10^−10^	0.31	1.00	rs11630054	0.0067	3.3×10^−10^	0.34	0.99
16q12.2	rs12598049	0.0074	3.0×10^−11^	0.32	0.99	rs6499550	0.007	8.2×10^−11^	0.32	0.98
16q22.2	rs11864453	0.0057	4.6×10^−08^	0.40	0.99	rs1035560	0.0058	1.2×10^−08^	0.40	0.99
17q21.2	rs7224737	0.0073	2.2×10^−09^	0.23	0.99	rs7224737	0.0068	5.2×10^−09^	0.24	1.00
17q25.1	rs10512597	0.0078	2.2×10^−08^	0.18	0.94	rs35489971	0.0077	1.6×10^−08^	0.18	0.94
20q13.12	rs1800961	0.0183	6.8×10^−09^	0.03	0.95	rs1800961	0.0178	1.7×10^−09^	0.03	0.99
21q22.2	rs4817986	0.0091	1.9×10^−14^	0.28	0.95	rs9808651	0.0093	5.4×10^−16^	0.28	0.94
22q13.33	rs6010044	0.0074	2.5×10^−08^	0.20	0.89	rs75347843	0.0082	4.3×10^−08^	0.19	0.76

Further detail about these loci and the lead variants is provided in S3 Table. *Abbreviations*: HapMap refers to the GWA study using imputation based on the HapMap project. 1000G refers to the GWA study using imputation based on the 1000 Genomes Project. Variants were coded according to the fibrinogen increasing allele. MAF refers to minor allele frequency.

### Non-overlapping loci

**Fig 2:**
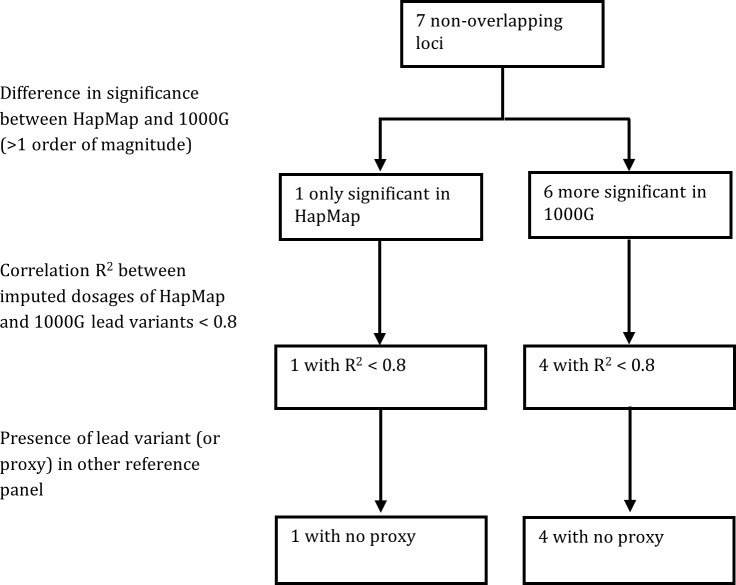
Summary of the differences between HapMap and 1000G imputation for the seven non-overlapping loci.

The lead variants for the seven non-overlapping loci always differed between the HapMap and 1000G GWA studies, and all *P*-value differences were greater than one order of magnitude (for example: from 5×10^−8^ to 5×10^−9^ or less). Differences between HapMap and 1000G imputation for the seven non-overlapping loci are summarized in Fig 2.

**Fig 3:**
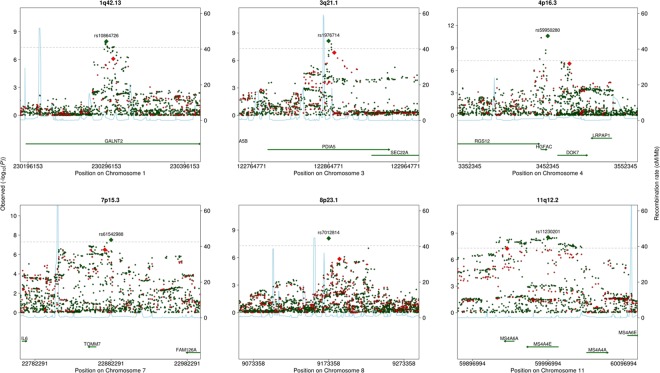
Regional plots of non-overlapping loci that were more significantly associated with fibrinogen in the 1000G GWA study, including variants from both the HapMap (red) and 1000G (green) GWA studies.

Regional plots of the six loci significant only in the 1000G GWA study are shown in Fig 3. For four of these six loci, the correlation r^2^ between allelic dosages of the most associated variants imputed using HapMap and 1000G was less than 0.8 (S4 Table). None of the 1000G lead variants among these four loci were included in the HapMap GWA study, and neither were any good proxies (S5 Table).

**Fig 4:**
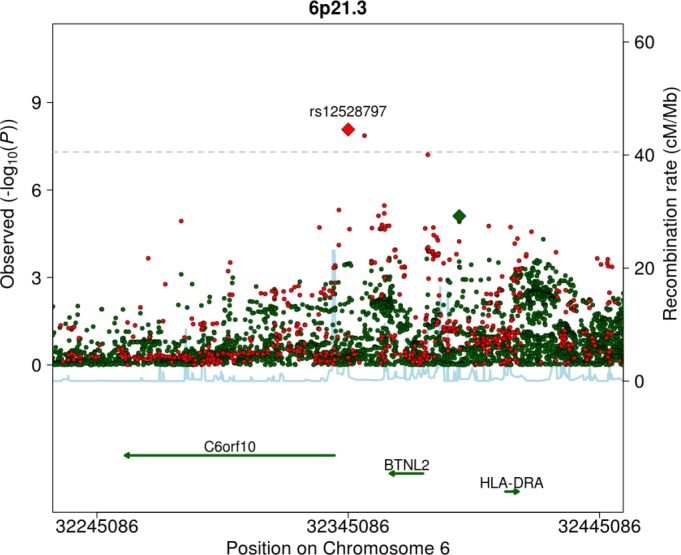
Regional plot of 6p21.3, a non-overlapping locus that was more significantly associated with fibrinogen in the HapMap GWA study, including variants from both the HapMap (red) and 1000G (green) GWA studies.

A regional plot of the 6p21.3 locus, which was significant only in the HapMap GWA study, is shown in Fig 4. The most significant *P*-value at the locus was 8.5×10^−9^ in the HapMap GWA study compared to 7.9×10^−6^ in the 1000G GWA study. The correlation r^2^ between imputed dosages of the HapMap and 1000G lead variants was low (0.07). The HapMap lead SNP was included in the 1000G GWA study under a different name, rs114339898, but the imputation quality was only sufficient for inclusion in seven of the studies (S5 Table).

### Overlapping loci

Regional plots of the 29 overlapping loci are shown in S4 Table. The lead variants of eight of the 29 overlapping loci were the same for the HapMap and 1000G GWA studies. *P*-value differences between the HapMap and 1000G GWA studies were often small: they were smaller than or equal to one order of magnitude for 22 loci. *P*-values differed by more than one order of magnitude for seven loci. Five of these loci were more significant in the 1000G GWA study (2q37.3, 4q31.3, 10q21.3, 12q24.12, and 21q22.2), while two of these loci were more significant in the HapMap GWA study (5q31.1 and 8q24.3).

Among the five overlapping loci with lower *P*-values in the 1000G GWA study, the correlation r^2^ between imputed dosages of lead variants from HapMap and 1000G was higher than 0.8 for 4 loci, but was 0.68 for the 12q24.12 locus (S4 Table). There was no good proxy of the 1000G lead variant at the 12q24.12 locus included in the HapMap GWA study.

**Fig 5:**
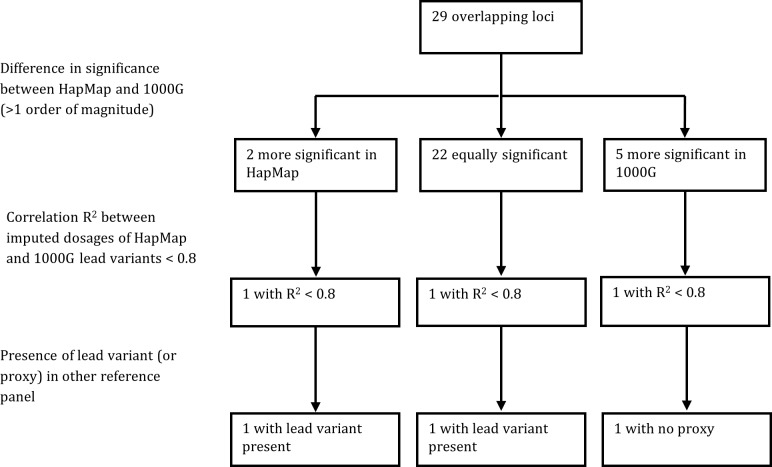
Summary of the differences between HapMap and 1000G imputation for the 29 overlapping loci.

The 5q31.1 and 8q24.3 loci had lower *P*-values in the HapMap GWA study. The correlation r^2^ between imputed dosages from HapMap and 1000G was almost perfect for 5q31.1, but was 0.75 for 8q24.3. The HapMap lead variant of the 8q24.3 locus was also included in the 1000G GWA study. These differences between HapMap and 1000G imputation for the 29 overlapping loci are summarized in Fig 5.

### Sensitivity analyses

Because more independent variants are included in the 1000G GWA study [20, 21], using the conventional genome-wide significance threshold of 5×10^−8^ may result in an increased type I error rate. When we used a more stringent genome-wide significance threshold of 2.5×10^-8^for the 1000G GWA study as suggested by Huang et al. [20], there were 4 loci significant only in the HapMap GWA study, 5 loci significant only in the 1000G GWA study, and 26 overlapping loci (Fig 1B). Three loci that were significant using both HapMap and 1000G imputation thus became non-significant when the stricter significance threshold was applied to the 1000G results.

Genomic inflation factors to correct for genomic control were calculated separately for the HapMap and 1000G analyses of each study. Thus, differences in the genomic inflation factors could explain some of the differences between the HapMap and 1000G results. When we repeated the HapMap and 1000G GWA study without applying genomic control corrections, 2 loci were associated only with circulating fibrinogen concentration in the HapMap GWA study, 6 were only associated in the 1000G GWA study, and 30 were associated in both GWA studies (Fig 1C and S6 Table).

For practical reasons, not all of the studies used the same imputation software, analysis software, or covariates for the HapMap and 1000G analyses. Specifically, fewer studies used principal components in the HapMap GWA study. When we restricted the analysis to those studies that used the same imputation software, analysis software, and covariates in the HapMap and 1000G GWA studies (S7 Table and S8 Table), 3 loci were associated only in the 1000G GWA study, and 6 were associated in both the HapMap and the 1000G GWA studies (Fig 1D and S9 Table). No loci were associated only in the HapMap GWA study.

## Discussion

In our fibrinogen GWA study of 91,953 individuals, using 1000G instead of HapMap imputation led to the identification of six additional fibrinogen loci, suggesting an improvement in the detection of associated signals. Nevertheless, there was also one locus that was only identified when using HapMap imputation, and the advantage of 1000G imputation was attenuated when using a more stringent Bonferroni correction for the 1000G GWA study. The inclusion of indels in the 1000G GWA study did not lead to the identification of any new loci. Only one locus in our 1000G GWA study was led by an indel, and it was in strong linkage disequilibrium with a SNP present in HapMap.

While this is the first study of the impact of HapMap and 1000G imputation on genome-wide associations using exactly the same individuals in a large-scale consortium setting, four previous studies have addressed this question on a smaller scale. In the Wellcome Trust Case Control Consortium, consisting of 2000 for seven diseases (bipolar disorder, coronary artery disease, Crohn's disease, hypertension, rheumatoid arthritis, type 1 and 2 diabetes) and 3000 shared controls, Huang et al. re-analyzed GWA studies of these seven diseases with 1000G imputation, and found two novel loci: one for type 1 diabetes and one for type 2 diabetes [20]. A more conservative genome-wide significance threshold of 2.5×10^−8^ was used in the 1000G GWA studies, while the MAF inclusion threshold was the same at 1%. The second study was a 1000G imputed GWA study of around 2000 cases of venous thrombosis and 2400 controls [22]. Using a conservative P-value threshold of 7.4×10^−9^, but no MAF threshold, Germain et al. identified an uncommon variant at a novel locus that was not identified in the HapMap GWA study [22]. Third, the National Cancer Institute Breast and Prostate Cancer Cohort Consortium found no new loci by applying 1000G imputation to their existing dataset of 2800 cases and 4500 controls [23, 24]. The conventional genome-wide significance threshold of 5×10^−8^ was used, but no MAF threshold was used. Fourthly, Wood et al. compared HapMap and 1000G imputation for a total of 93 quantitative traits in 1210 individuals from the InCHIANTI study [25]. Using a significance threshold of 5×10^−8^ for both the HapMap and 1000G GWA studies, they found 20 overlapping associations, 13 associations that were only significant using 1000G imputation, and one association that was only significant using HapMap imputation. For the association significant only in HapMap, the *P*-value difference between HapMap and 1000G lead variants was less than one order of magnitude. When the authors lowered their significance threshold to 5×10^−11^ to reflect the number of tests being done in analyzing multiple traits, 9 associations remained significant based on HapMap imputation and 11 associations remained significant based on 1000G imputation.

All four of these comparison studies used an earlier 1000 genomes reference panel. The present study adds to the literature as it is based on the widely implemented Phase 1 Version 3 of 1000G. Crucially, the large sample size allowed us to examine differences at many non-overlapping and overlapping loci, and improved the generalizability of our results, as ongoing GWA studies are often conducted in large consortia.

Two further studies with different approaches also provide insights. First, Springelkamp et al. found a novel locus using 1000G imputation even though the sample size was smaller than the previous HapMap GWA study [26, 27]. The same genome-wide significance (5×10^−8^) and MAF (1%) thresholds were used. The lowest *P*-value at the locus was 1.9×10^−8^. Because different individuals were included in these GWA studies, the difference between HapMap and 1000G may partially be explained by sampling variability. Second, Shin et al. identified 299 SNP-metabolite associations based on HapMap imputation, and reexamined the associated loci using 1000G imputation in the same individuals [28]. They found that HapMap and 1000G imputation yielded similar *P*-values and variance explained for all but one loci. For that locus, the 1000G imputation based association was considerably stronger: the explained variance increased from 10% to 16%, and the *P*-value decreased from 8.8×10^−113^ to 7.7×10^−244^. Although Shin et al. did not compare loci identified using HapMap and 1000G, their results do support our finding that large differences in association strengths are possible, albeit not at every locus. All these studies, along with the current study, suggest that additional signals not previously identified in HapMap GWA studies can be found using the 1000G GWA study, with the same sample size.

In the current study we demonstrate that, although 1000G imputation was overall more effective at identifying associated loci, HapMap imputation may outperform 1000G imputation for specific loci. The 6p21.3 locus, corresponding to the major histocompatibility complex (MHC), was significant in the HapMap GWA study but not in the 1000G GWA study. The MHC locus is highly polymorphic and hosts many repetitive sequences, rendering it difficult to genotype and sequence [29–31]. The HapMap reference panel was based largely on the genotyping of variants that were known at that time, whereas the 1000G reference panel is based entirely on low-coverage sequencing. This may explain the rather large discrepancy between HapMap and 1000G at this locus.

Differences in associations when GWA studies are based on different participants can be explained by sampling variability, even with the same sample size. Hence, by using exactly the same participants in the HapMap and 1000G comparisons in the present project, we rule out both statistical power and sampling variability as possible explanations for differences between the HapMap and 1000G GWA studies. Several real differences between the HapMap and 1000G reference panels may underlie the net benefit of 1000G imputation. The HapMap reference panel was largely based on genotypes of known variants, whereas the 1000G reference panel was primarily based on low-pass whole genome sequencing, enhancing the inclusion of novel variants. Additionally, most studies used only a small number of European-ancestry participants for HapMap imputation, whereas they used a larger number of participants of all available ancestries for 1000G imputation, introducing further haplotypes into the imputation process.

Nevertheless, some analytical differences between the HapMap and 1000G analyses were not controlled for in our main analysis and therefore remain as potential alternative explanations. First, genomic control corrections were applied to the results of each of the studies before meta-analysis, separately for the HapMap and 1000G GWA studies. As a result, for any given study, there could be differences between the correction applied to the HapMap GWA analysis and to the 1000G GWA analysis. As these differences do not appear to differ systematically between the HapMap and 1000G GWA analyses in our study, the genomic control corrections are unlikely to explain our results. The results from our sensitivity analysis were concordant with this interpretation: when no genomic control corrections were applied there were 6 loci only significant in the 1000G GWA study compared to 2 loci only significant in the HapMap GWA study.

The second difference between the HapMap and 1000G GWA studies that may explain our findings is that in the 1000G GWA study more studies were adjusted for ancestry-informative principal components. This difference reflects common practice, as population stratification is suspected to have a stronger influence on variants with lower MAF, and 1000G includes more of these [32]. However, the adjustments are applied to variants across the spectrum of minor allele frequencies, which may have influenced our results.

Thirdly, some studies used different software for HapMap and 1000G imputation (S1 Table). The imputation quality metrics used by IMPUTE and MACH differ, and this has traditionally been dealt with by applying different imputation quality thresholds: > 0.3 for MACH and > 0.4 for IMPUTE [5, 33]. In studies that used different imputation software for the HapMap and 1000G GWA studies, the filtering of variants can therefore differ. There may, additionally, be real differences in imputation quality. Finally, some studies used different analysis software (S3 Table). When we restricted our analysis to only those studies that used the same covariates, analysis software, and imputation software for the HapMap and 1000G GWA studies, 3 loci were only significant in the 1000G GWA study, while all loci significant in the HapMap GWA study were also significant in the 1000G GWA study. This suggests that differences in imputation software, analysis software, and covariates do not fully explain the observed difference between the HapMap and 1000G GWA studies, and that there are real differences resulting from choice of reference panel.

1000G GWA studies include more independent statistical tests than HapMap GWA studies [20, 21]. Thus, while a *P*-value threshold of 5×10^−8^, correcting for 1 million independent tests, maintains the type I error rate at 5% for HapMap GWA studies, this may not be the case for 1000G GWA studies. Using 1000G pilot data, Huang et al. estimated that 2 million independent tests were being done, and thus suggested a *P*-value threshold of 2.5×10^−8^ [20]. In our study we used a *P*-value threshold of 5×10^−8^ for both the HapMap and 1000G GWA studies, in accordance with the majority of published 1000G GWA studies [26, 34–37]. When we used the threshold of 2.5×10^−8^ in the 1000G imputed GWA study, the difference between the HapMap and 1000G GWA studies became smaller. Thus, while we expect applying 1000G imputation may lead to novel findings using the conventional genome-wide significance threshold, this expectation may not be met when using stricter, and perhaps more appropriate thresholds. In other words, using the traditional significance threshold for 1000G may increase the type 1 error rate, which may account for some additional significant loci detected in 1000G GWA studies.

In this study we only examined variants with a MAF of greater than 1%. This restriction was common practice for HapMap GWA studies, but given the improved coverage of rare variants in 1000G, this may not remain the case for 1000G GWA studies. Different MAF thresholds have been used in published 1000G GWA studies, although many have used 1% [20, 22, 23, 26, 27, 34–40]. Therefore, an advantage of 1000G not illustrated by this study may be the identification of rare variants, at new loci or as secondary signals at known loci. The advantage of 1000G imputation will then in part depend on the importance and impact of rare variants in the trait being studied, as well as the distribution of these variants. Rare and uncommon variants are often clustered in genes with previously associated common variants, limiting the new biology revealed through their identification [41, 42]. This appears to be the case for fibrinogen concentration as well [43, 44].

In conclusion, we show that the reference panel used in GWA studies can have an impact on the identification of common variants, although our results do not support the expectation that 1000G imputation always outperforms HapMap imputation, as we found one locus that appeared to be better covered in HapMap. This suggests that GWA studies will continue to be more successful as newer reference panels such as the Haplotype Reference Consortium are adopted. Nevertheless, our results also suggest that the benefits of 1000G are considerably reduced when the additional independent tests introduced by 1000G imputation are corrected for. Given that the bulk of the new information provided by 1000G imputation relates to low-frequency variants, we expect the penalty increased multiple testing burden to become less relevant in future studies as the power to examine these low-frequency variants increases with larger sample sizes and enhanced imputation quality. Imputation using the Haplotype Reference Consortium reference panel improves the imputation quality of low-frequency variants when compared to 1000G, and future reference panels based on the wealth of whole-genome sequencing data currently being generates by efforts such as TOPMed are likely to continue this trend [45].

## Methods

### Population

The sample for both the HapMap and 1000G GWA studies consists of 22 studies including the same 91,953 European-ancestry participants. The sample is largely a subset of the sample used in our previous work, and when possible the same analyses were used in this project [44, 46]. However, to ensure that only the same individuals were used, one or both of the analyses was rerun using only overlapping individuals when necessary. All studies were approved by appropriate research ethics committees and all respondents signed informed consent prior to participation. The ARIC study was approved by the University of Mississippi Medical Center IRB, Wake Forest University Health Sciences IRB, University of Minnesota IRB, and John Hopkins University IRB. The B58C study was approved by the South East England Multi-Centre Research Ethics Committee and the London & South East Committee of the National Research Ethics Service. The BMES was approved by the University of Sydney and the Western Sydney Area Health Service Human Research Ethics Committees. The CHS was approved by the Wake Forest University Health Sciences IRB, University of California, Davis IRB, John Hopkins University IRB, and University of Pittsburgh IRB, and University of Washington IRB. The FHS was approved by the Bostin University IRB. The GHS was approved by the Ethics Committee of the Landesärztekammer Rheinland-Pfalz (State Chamber of Physicians of Rhineland-Palatinate, Germany). The GOYA-Male study was approved by the regional scientific ethics committee of Copenhagen, Denmark, and the Danish data protection board. The HCS was approved by the University of Newcastle and Hunter New England Human Research Ethics Committee. The InCHIANTI study was approved by the Italian National Institute of Research and Care of Aging Institutional Review and Medstar Research Institute (Baltimore, MD). The LBC1921 study was approved by the Lothian Research Ethics Committee and the Scotland A Research Ethics Committee. The LBC1936 study was approved by the Multi-Centre Research Ethics Committee for Scotland and the Lothian Research Ethics Committee and the Scotland A Research Ethics Committee. The LURIC study was approved by the Ethics Committee at the Ärztekammer Rheinland-Pfalz. The NTR study was approved by the Medical Ethical Committee of the VU University Medical Center Amsterdam, and the Central Committee on Research Involving Human Subjects of the VU University Medical Center Amsterdam. The PROCARDIS study was approved by the Ethics Committee of the Karolinska Institutet. The PROSPER-PHASE study was approved by the Greater Glasgow Community/Primary Care Local Research Ethics Committee, Dumfries and Galloway Health Board Local Research Ethics Committee, Argyll and Clyde Health Board Local Research Ethics Committee, Lanarkshire Research Ethics Committee, Research Ethics Committee of the Cork Teaching Hospitals, and the Medical Ethical Committee of the Leiden University Medical Center. The RS was approved by the Medical Ethics Committee of the Erasmus MC and the Dutch Ministry of Health, Welfare and Sport. The SardiNIA study was approved by the Ethics Committee at Azienda Sanitaria Locale (ASL) n°1 of Sassari, Sardinia, Italy. The SHIP was approved by the Medical Ethics Committee of the University of Greifswald. The TwinsUK study was approved by the NRES Committee London-Westminster (formerly St Thomas' Ethics Committee). The WGHS was approved by Brigham and Women’s Hostpital IRB.

### Genotyping and imputation

Genotyping and pre-imputation quality control methods for each study are shown in S7 Table. Studies imputed dosages of genetic variants using reference panels from the 1000 genomes project with MACH [47, 48] or IMPUTE [49]. Studies imputed variant dosages using Phase 2 reference panels from the HapMap project with MACH [47, 48], IMPUTE [49], or BIMBAM [50]. We excluded variants with MACH imputation quality < 0.3, IMPUTE/BIMBAM imputation quality < 0.4, or MAF < 0.01 from each study.

### Fibrinogen measurement

Fibrinogen concentration was measured in citrated or EDTA plasma samples using a variety of methods including the Clauss method, immunonephelometric methods, immunoturbidimetric methods, and other functional methods. Fibrinogen concentration was measured in g/L and natural log transformed. Details about the fibrinogen measurement are shown in S10 Table.

### Genome-wide association analysis

All analyses were adjusted for age and sex, and study specific covariates such as center or case/control status. In family studies, linear mixed models were used to account for family structure. Some studies adjusted the analysis for principle components to account for population structure and cryptic relatedness. Some studies used a different number of principle components in the HapMap and 1000G analyses. The adjustments and analysis software used by each study are shown in S8 Table. We applied a genomic control correction to the results of each of the studies before meta-analysis to remove any remaining genomic inflation. The genomic inflation factor used in this correction was calculated separately in the HapMap and 1000G analyses for each study. We meta-analyzed the results using an inverse-variance model with fixed effects implemented in METAL [51]. Loci were defined as the 500 Kb area on either side of lead variants (the variant with the smallest *P*-value). Build 36 positions of HapMap SNPs were converted to build 37 using the UCSC genome browser (http://genome.ucsc.edu/cgi-bin/hgLiftOver). Variants were annotated to genes using ANNOVAR version 2013Mar07. At the meta-analysis level, the imputation quality of each variant was defined as the sample-size weighted mean imputation quality across the studies, not including studies where the variant was filtered out.

### Comparison of HapMap and 1000G

When a locus was significant in both the HapMap and 1000G GWA studies we defined it as an overlapping locus. When a locus was significant in only one of the two analyses we defined it as a non-overlapping locus. To compare the strength of association in the HapMap and 1000G GWA studies, we identified loci with *P*-value differences of 1 order of magnitude or greater (for example: from 5×10^−8^ compared to 5×10^−9^ or less).

For each significant locus we used two approaches to assess the relationship between lead variants from HapMap and 1000G. First, we determined whether or not the more significant of the two lead variants or a good proxy (linkage disequilibrium r^2^ > 0.8) was included in the analysis of the other reference panel. If so, we examined its association in the other reference panel. Thus, if a locus was more significant in the 1000G GWA study, we checked whether the 1000G lead variant or a proxy was included in the HapMap GWA study. Second, we examined the correlation R^2^ between HapMap and 1000G lead variants in the form of imputed genotype dosages. This was performed for 5966 individuals from the Rotterdam Study (see study description in S1 Text) [52].

### Sensitivity analysis

First, we compared the results of the HapMap and 1000G GWA studies when applying a stricter Bonferroni-corrected *P*-value threshold of 2.5×10^−8^ to the 1000G GWA study. This threshold was suggested by Huang et al. to keep the type 1 error rate at 5% when using 1000G data [20]. Second, we repeated the analysis without using genomic control corrections. Third, we repeated the analysis in 34,098 participants using only the 10 studies that used the same imputation and analysis software as well as the same covariates for the HapMap and 1000G GWA studies.

## Supporting Information

S1 Fig Quantile-Quantile (QQ) plots comparing the HapMap and 1000G GWA studies. (DOCX)

S2 Fig Manhattan plot comparing the HapMap (red) and 1000G (green) GWA studies. (DOCX)

S3 Fig Comparison of lead variants of the HapMap and 1000G GWA studies of significant loci. (DOCX)

S4 Fig Regional plots of overlapping signals that were significant in both the HapMap (red) and 1000G (green) GWA studies. (DOCX)

S1 Table Characteristics of the included studies and their participants. (XLSX)

S2 Table Genomic inflation factors by study and imputation panel. (XLSX)

S3 Table Annotation of loci significant in the HapMap GWA study, 1000G GWA study, or both. (XLSX)

S4 Table Correlation between the lead variants from the HapMap and 1000G GWA studies. (XLSX)

S5 Table Differences between HapMap and 1000G for loci with a correlation R2 < 0.8 between imputed dosages of the HapMap and 1000G lead variants. (XLSX)

S6 Table Loci that were significant in either the HapMap or 1000G GWA studies with genomic control corrections. (XLSX)

S7 Table Genotyping and imputation methods of the included studies. (XLSX)

S8 Table Analysis software and covariates used by the included studies. (XLSX)

S9 Table Loci that were significant in either the HapMap or 1000G GWAS excluding studies that did not use the same imputation software, analysis software, or covariates. (XLSX)

S10 Table Sample and array type used for the fibrinogen measurement in each of the included studies. (XLSX)

S1 Text Supplementary Methods. (DOCX)
