# Supplementary figures and images for: Comparison of HapMap and 1000 Genomes Reference Panels in a Large-Scale Genome-Wide Association Study
Source: PLoS One. 2017 Jan 20;12(1):e0167742. doi: 10.1371/journal.pone.0167742 (PMC5249120; doi:10.1371/journal.pone.0167742)

**S1 Fig:** Quantile-Quantile (QQ) plots comparing the HapMap and 1000G GWA studies.


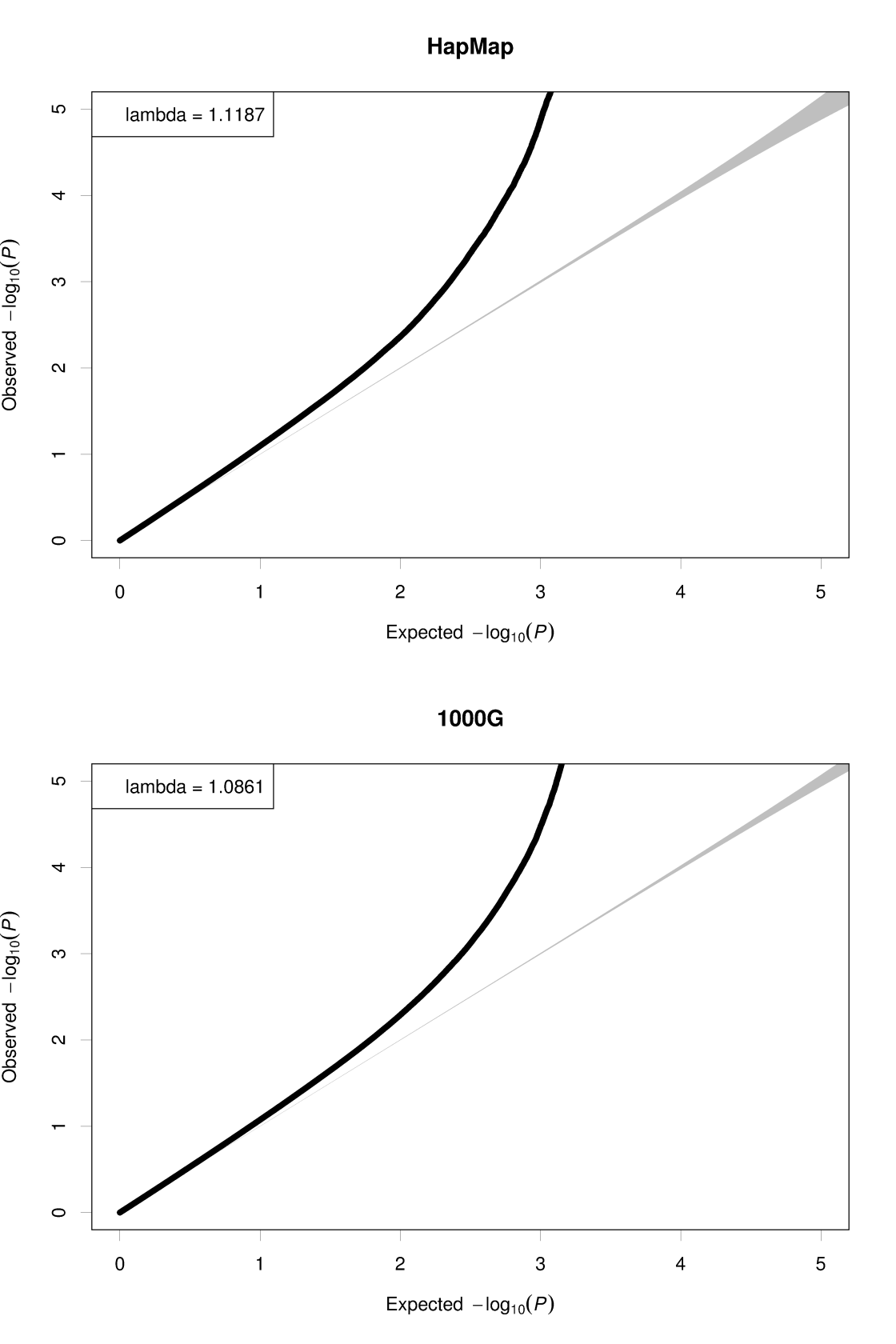

Supplement: S1 Fig — (DOCX) [file pone.0167742.s001.docx]

**S3 Fig:** Comparison of lead variants of the HapMap and 1000G GWA studies of significant loci.

**
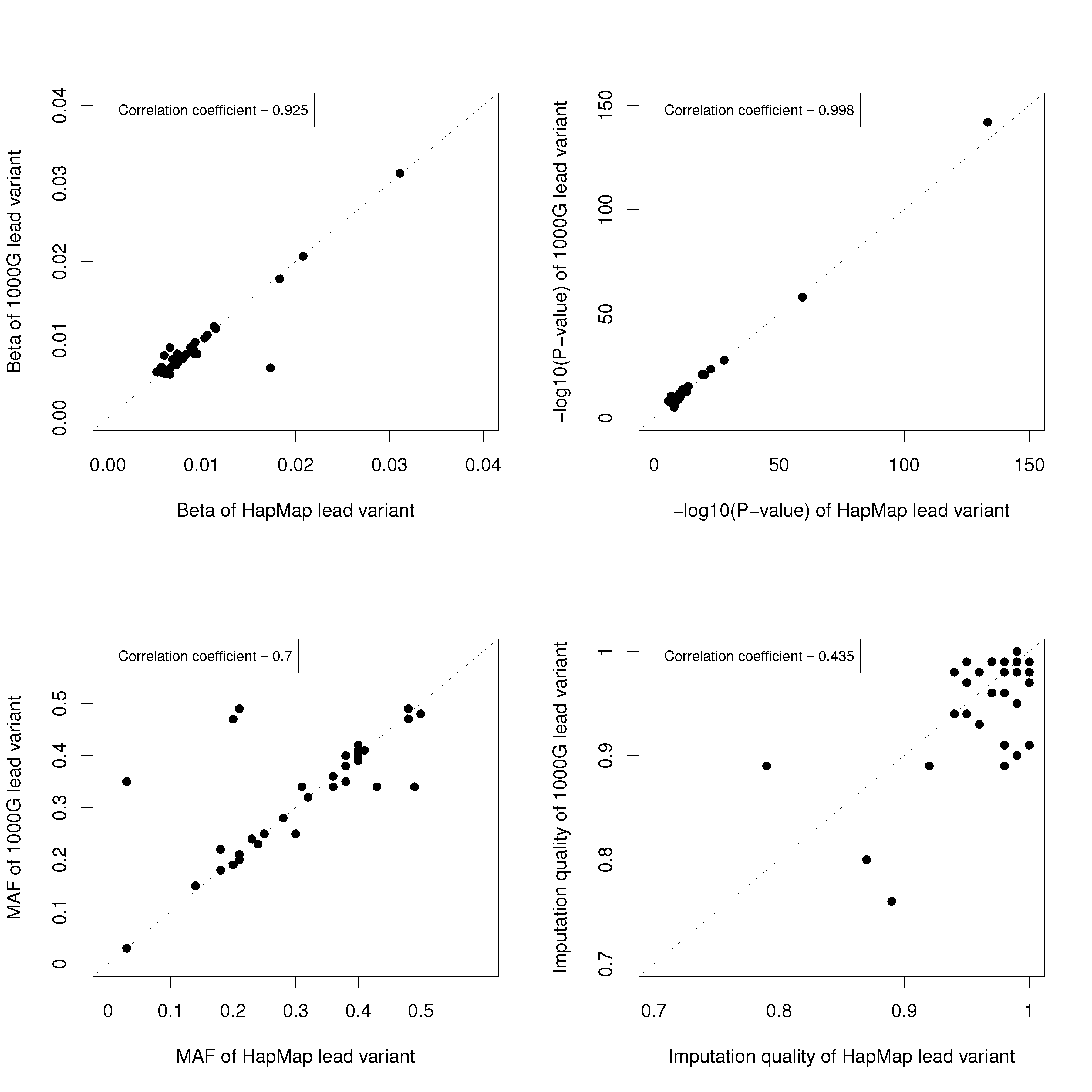
**

Supplement: S3 Fig — (DOCX) [file pone.0167742.s003.docx]
