# Supplementary material for: Comparison of HapMap and 1000 Genomes Reference Panels in a Large-Scale Genome-Wide Association Study
Source: PLoS One. 2017 Jan 20;12(1):e0167742. doi: 10.1371/journal.pone.0167742 (PMC5249120; doi:10.1371/journal.pone.0167742)
Supplement: S2 Fig — (DOCX) [file pone.0167742.s002.docx]

**S2 Fig:** Manhattan plot comparing the HapMap (red) and 1000G (green) GWA studies.*


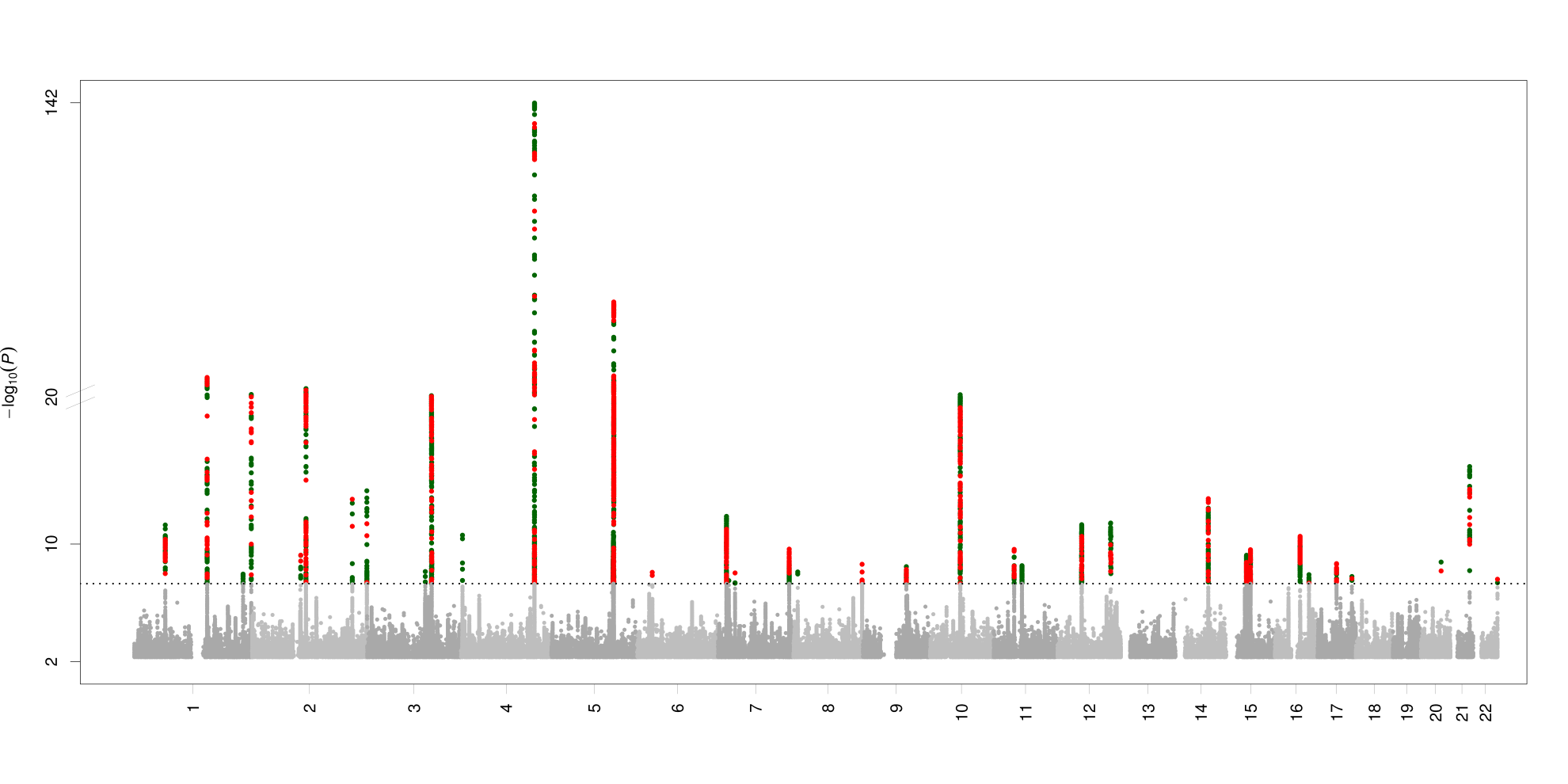


*Associations from the HapMap GWA study were plotted on top of associations from the 1000G GWA study, and were thus given priority when competing for space in the figure.
