# Supplementary material for: Comparison of HapMap and 1000 Genomes Reference Panels in a Large-Scale Genome-Wide Association Study
Source: PLoS One. 2017 Jan 20;12(1):e0167742. doi: 10.1371/journal.pone.0167742 (PMC5249120; doi:10.1371/journal.pone.0167742)
Supplement: S4 Fig — (DOCX) [file pone.0167742.s004.docx]

**S4 Fig:** Regional plots of overlapping signals that were significant in both the HapMap (red) and 1000G (green) GWA studies.


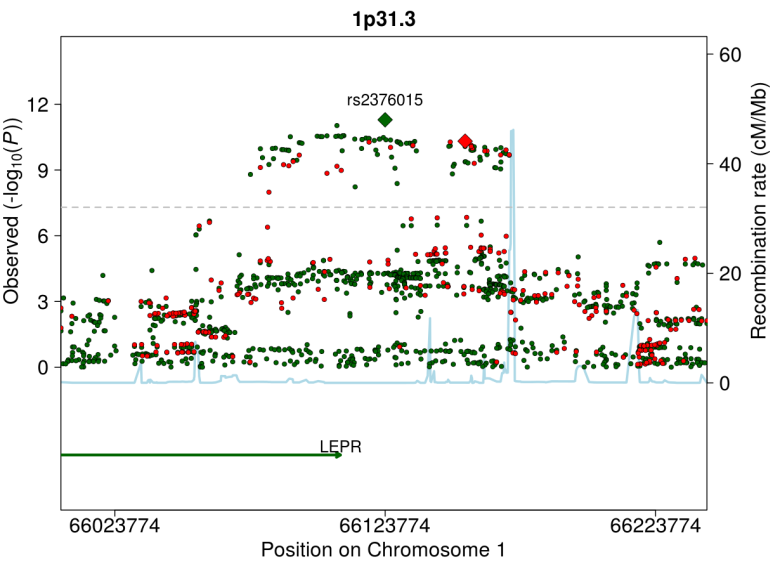
**
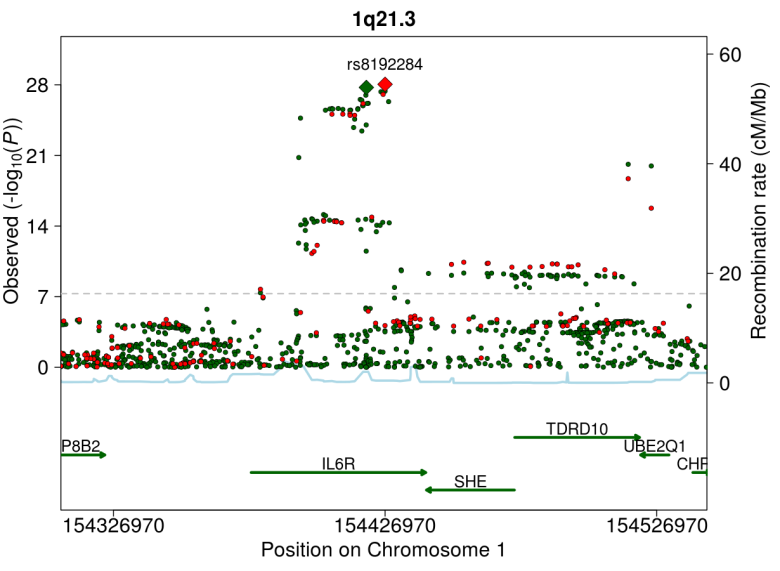
**


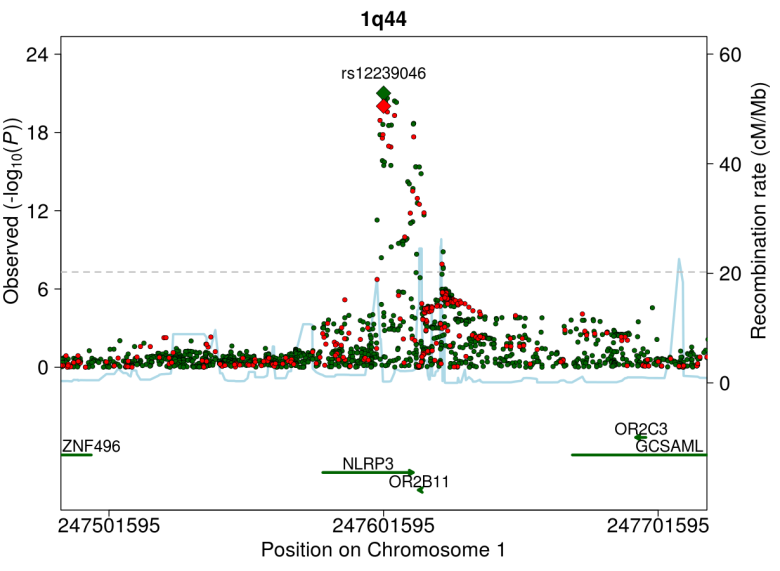

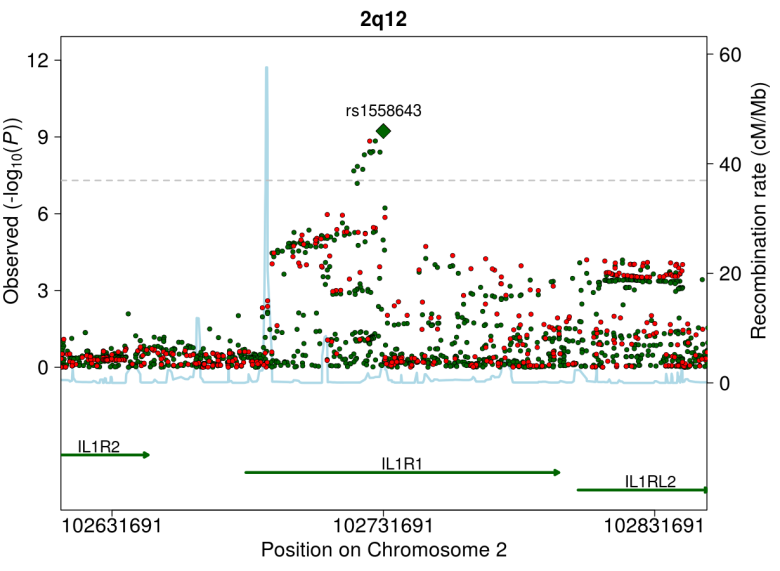


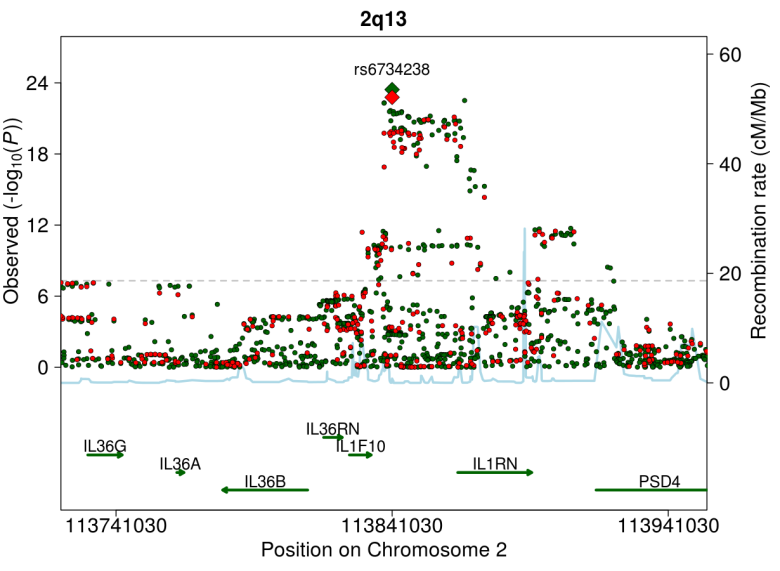

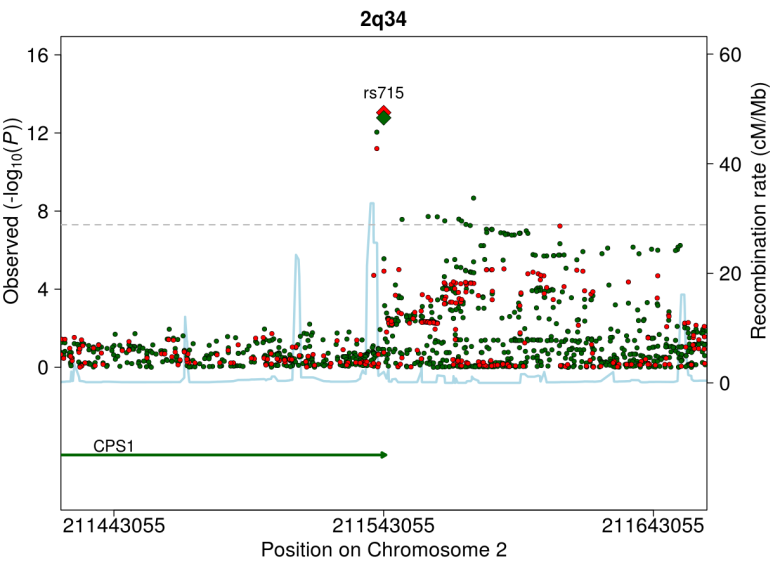


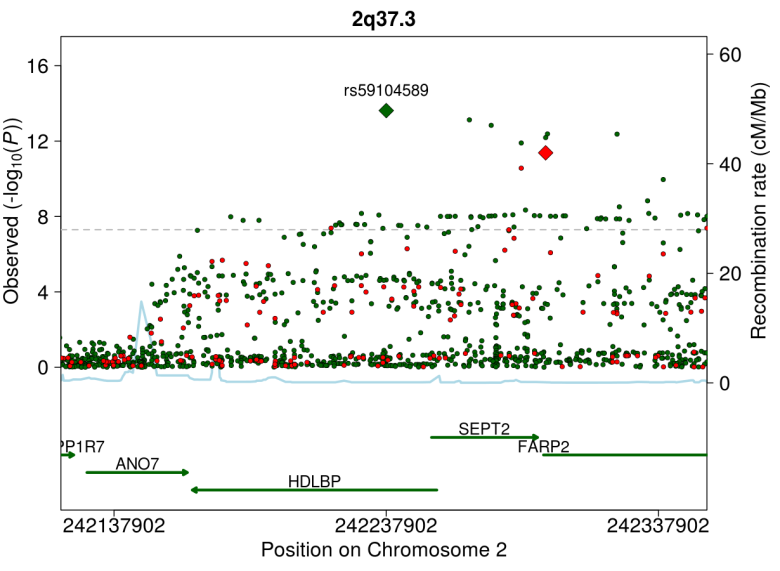

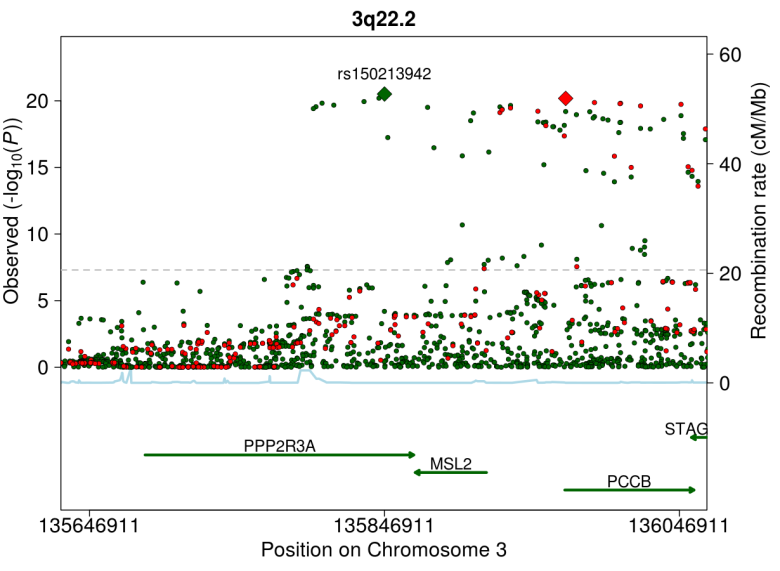


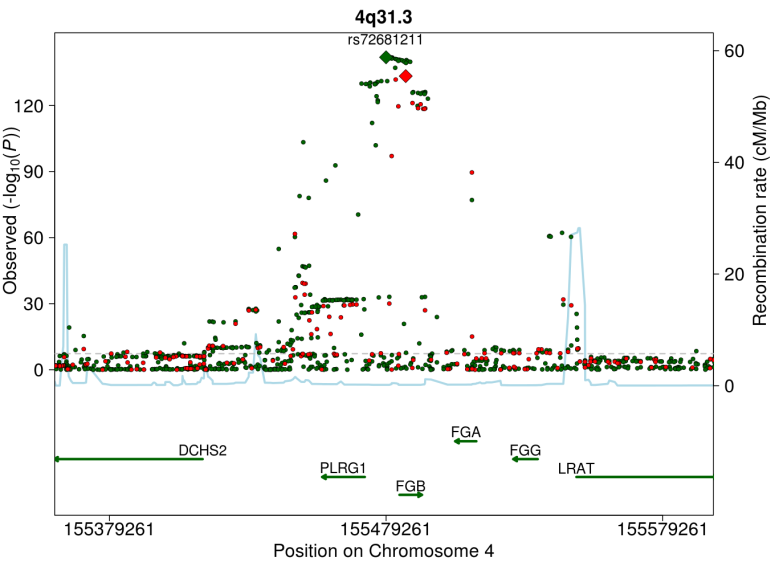

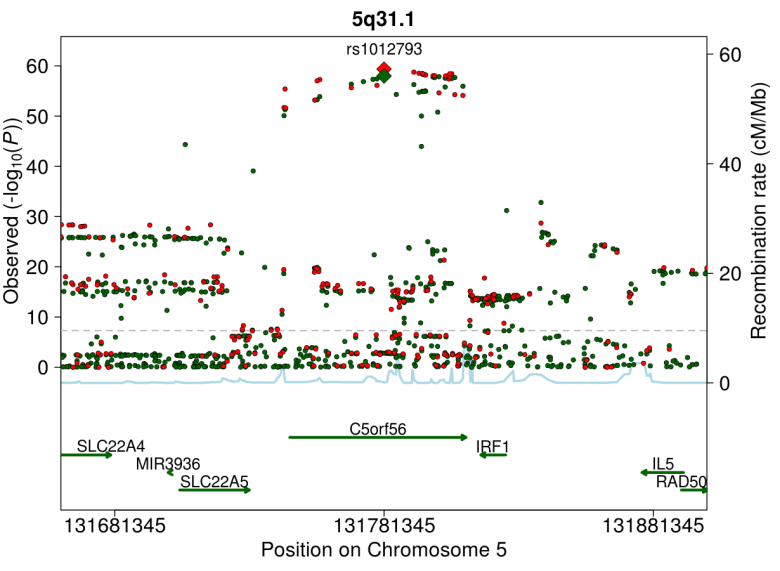


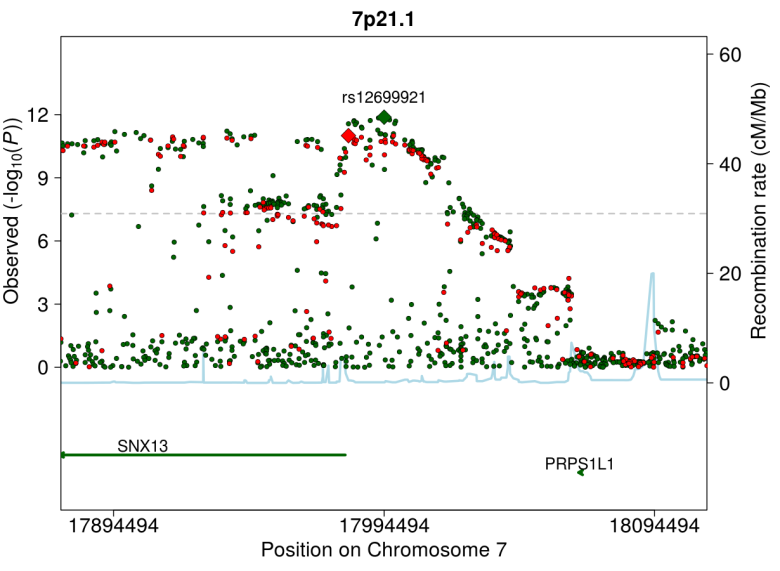

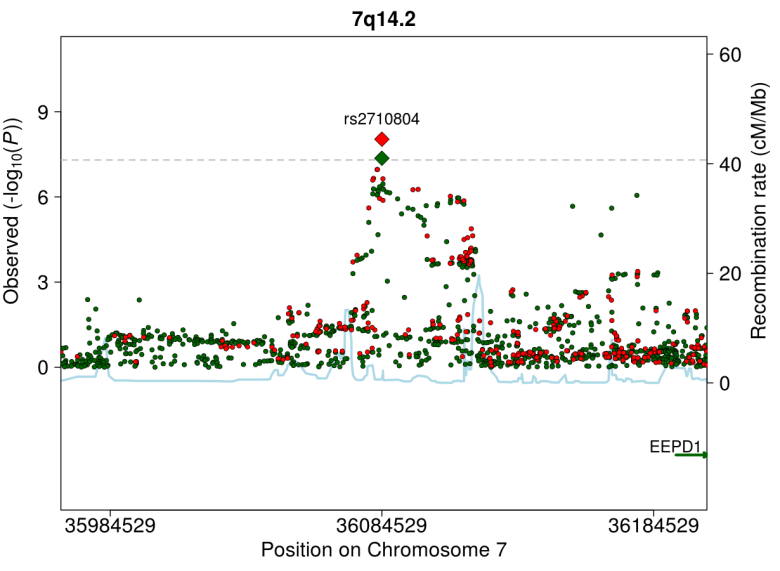


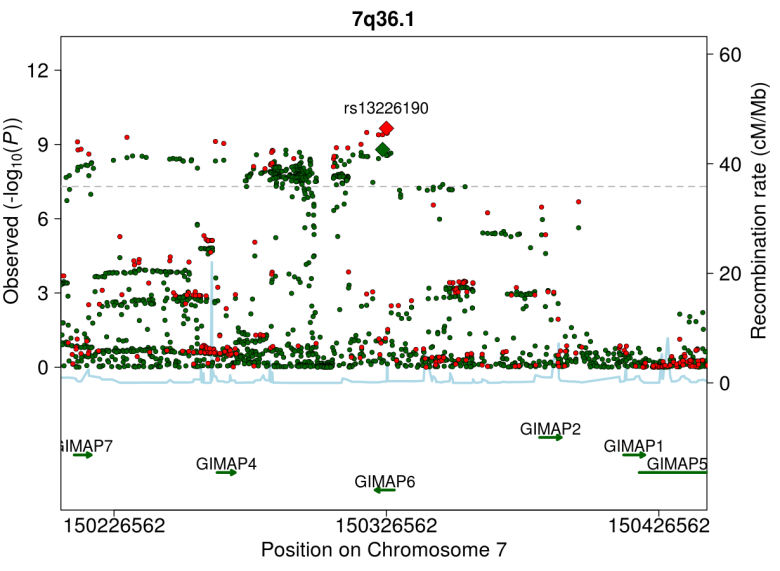

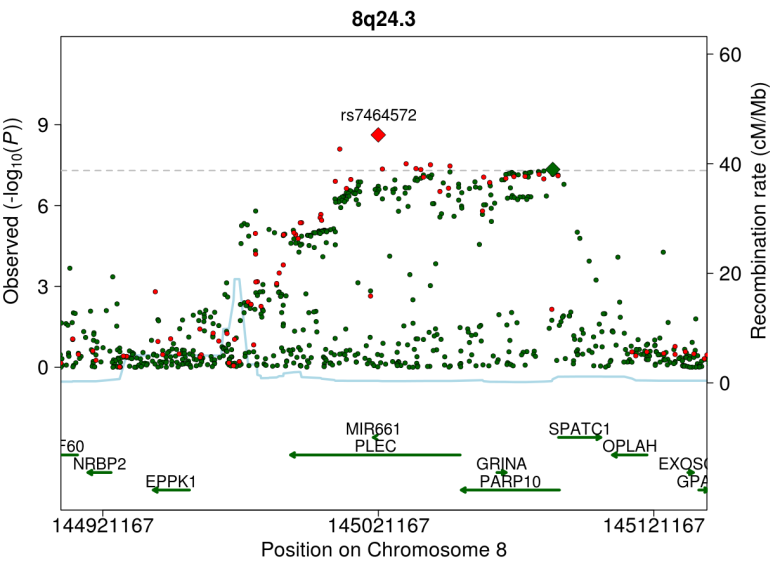


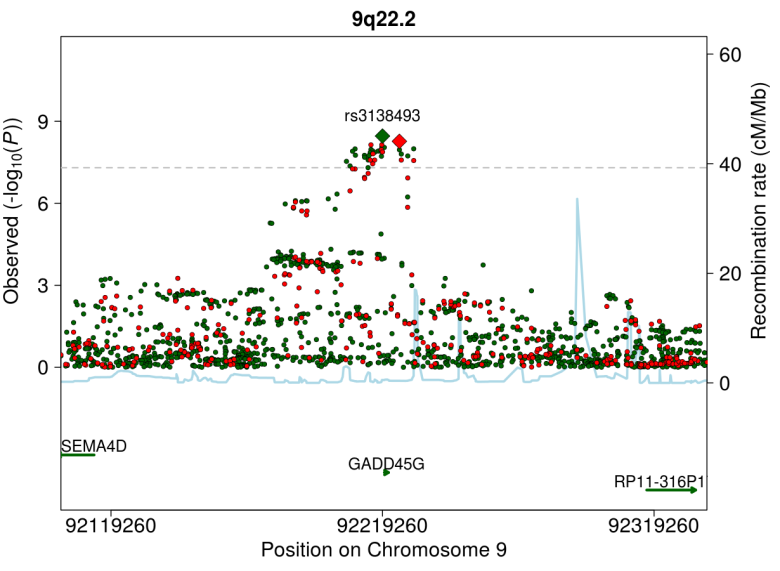

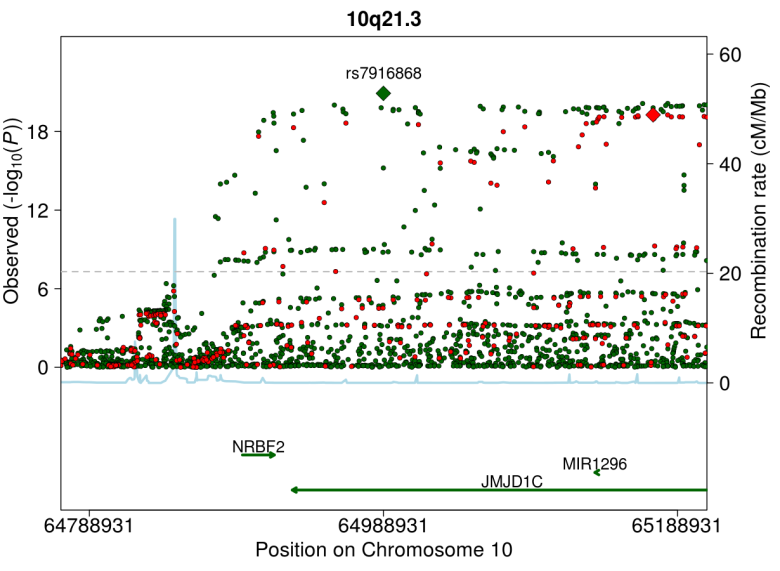


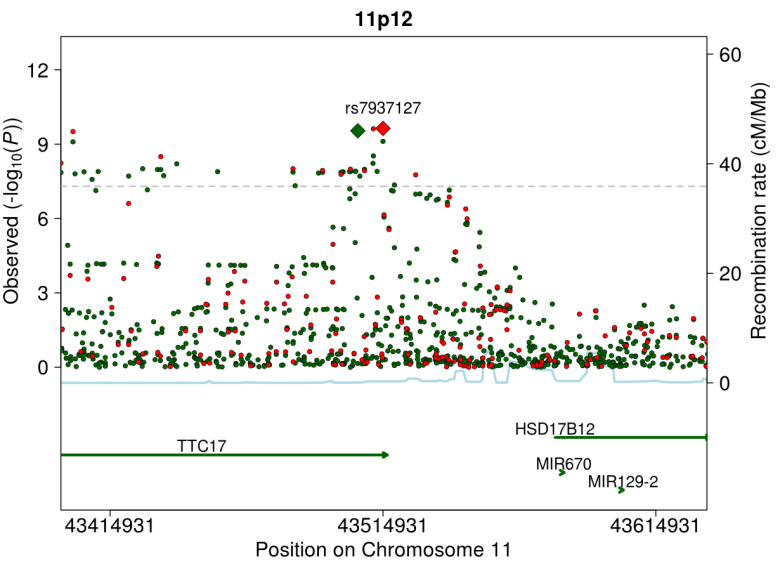

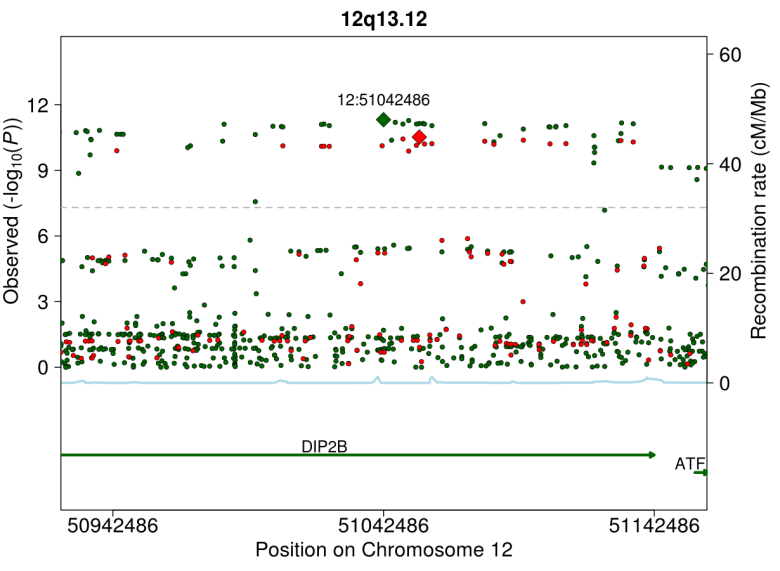


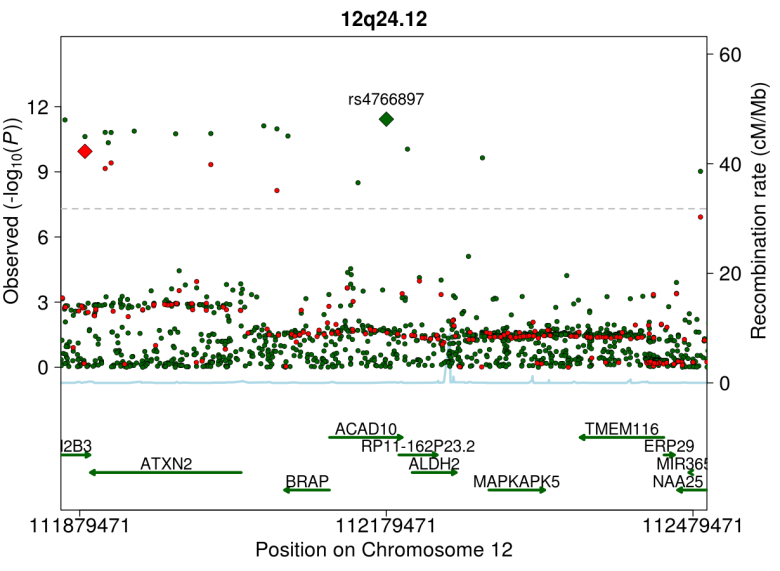

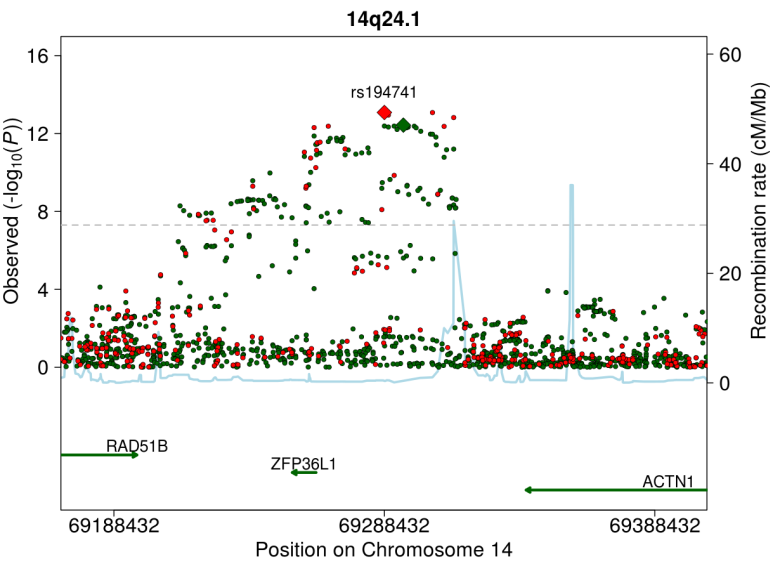


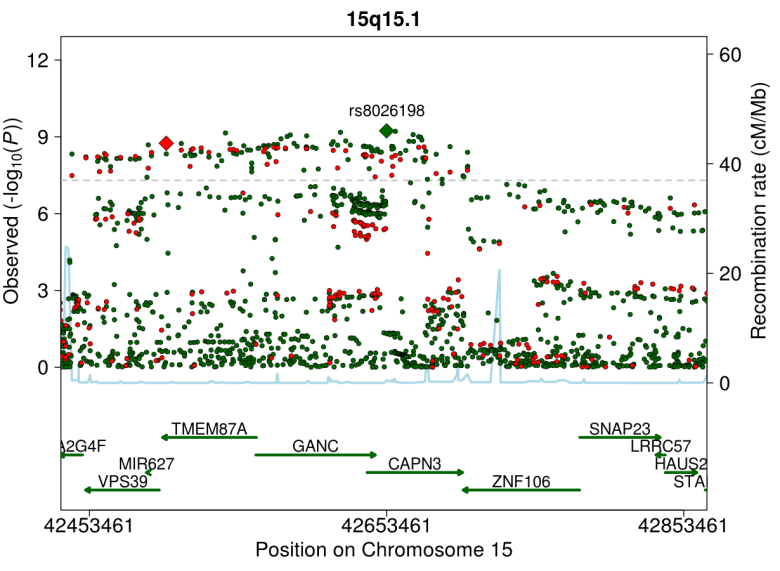

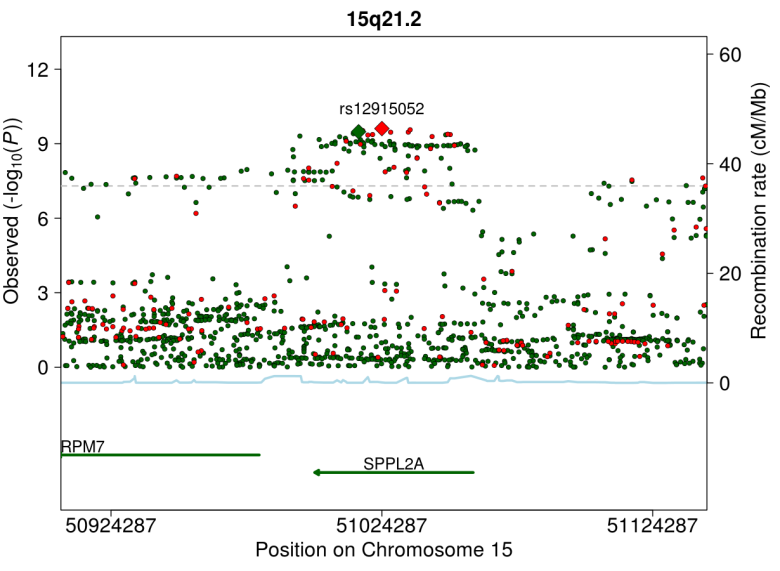


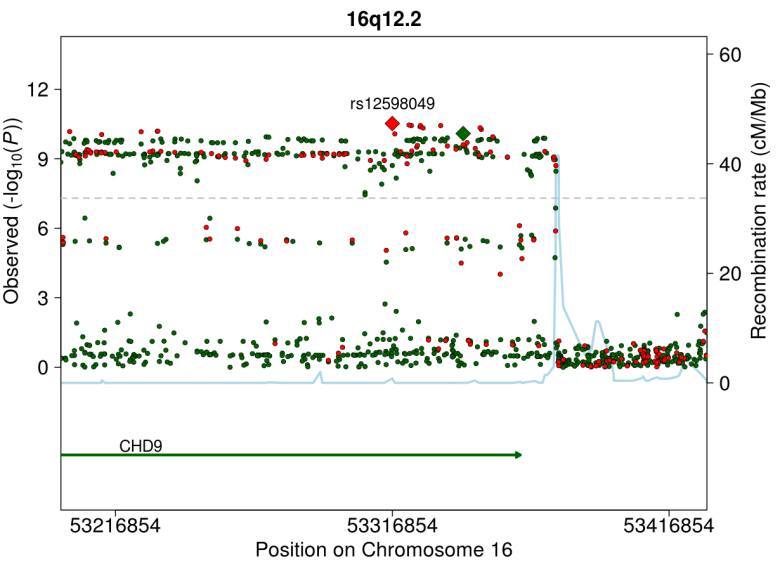

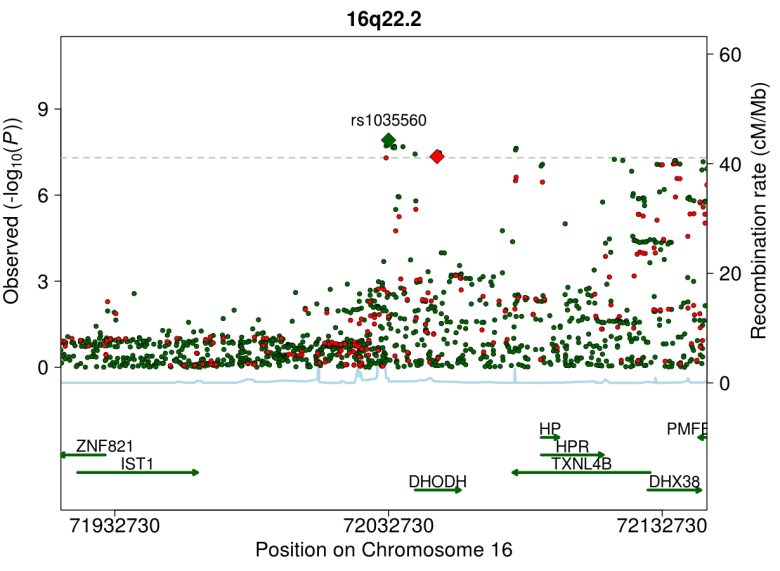


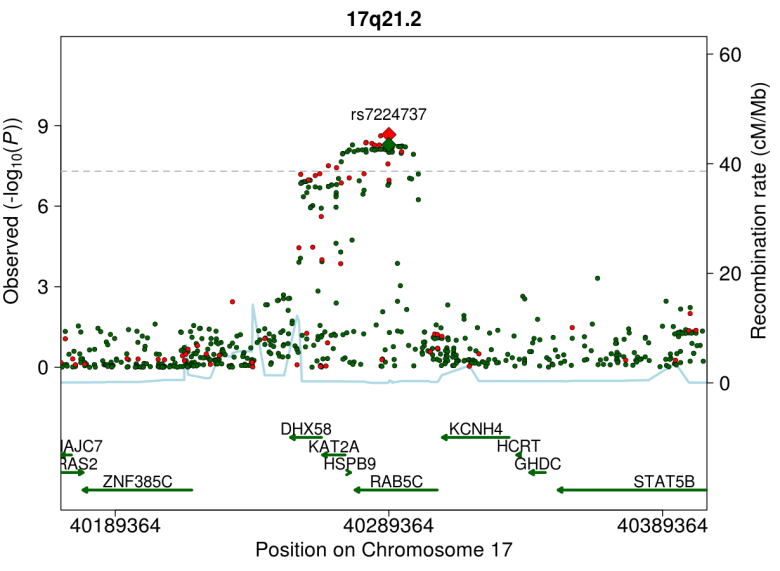

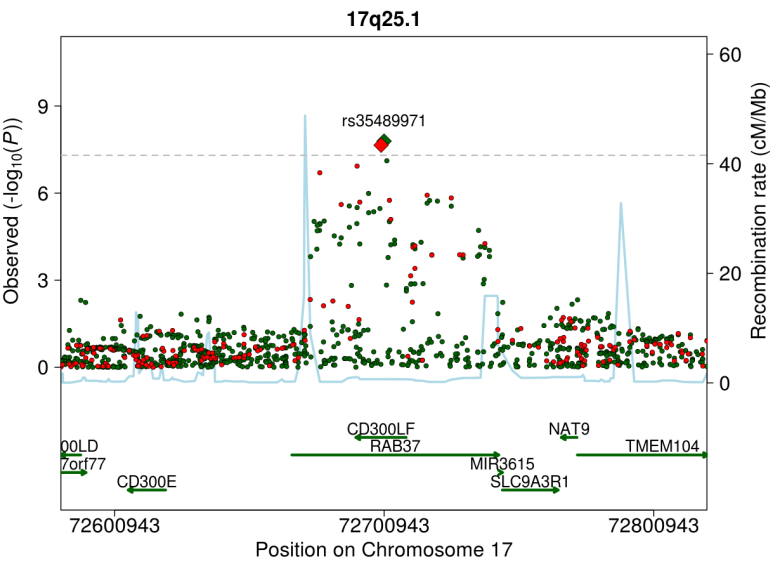


**
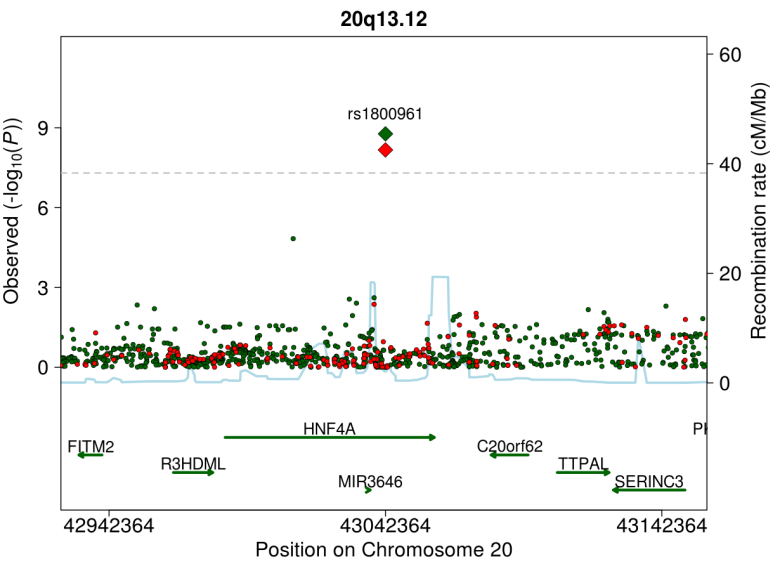

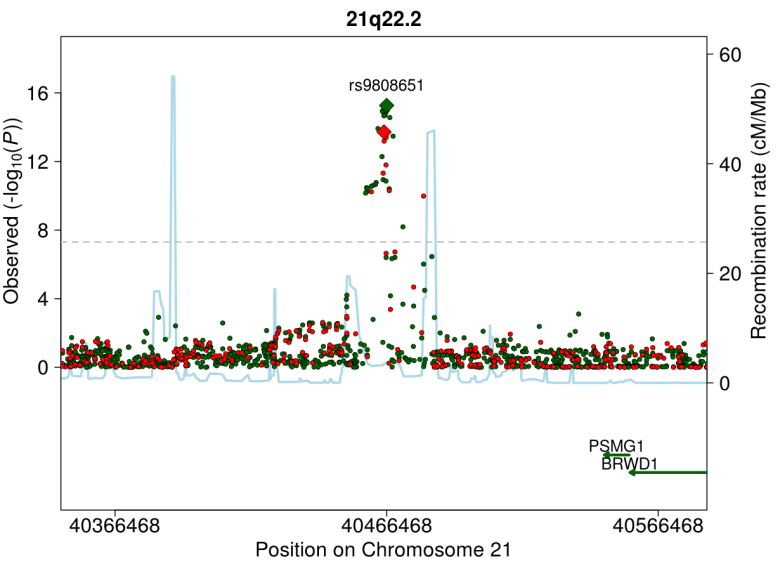
**

**
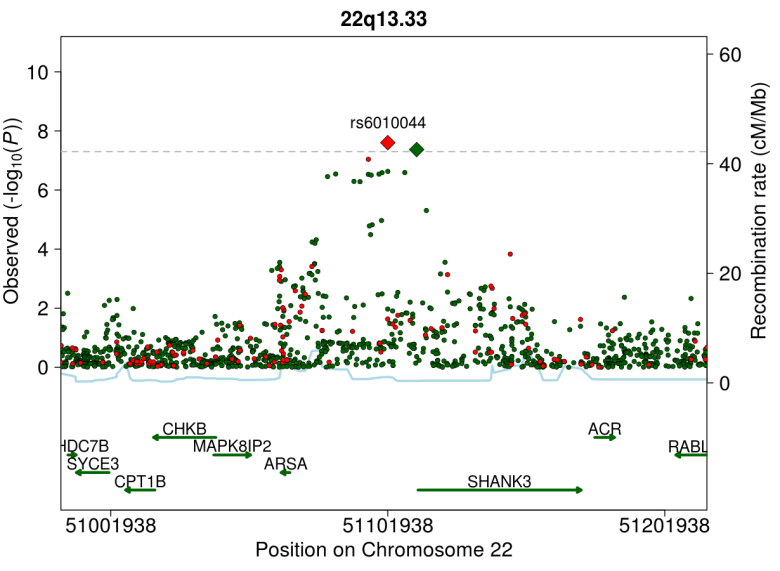
**
