# Supplementary material for: Comparison of HapMap and 1000 Genomes Reference Panels in a Large-Scale Genome-Wide Association Study
Source: PLoS One. 2017 Jan 20;12(1):e0167742. doi: 10.1371/journal.pone.0167742 (PMC5249120; doi:10.1371/journal.pone.0167742)
Supplement: S1 Text — (DOCX) [file pone.0167742.s015.docx]

**S1 Text:** Supplementary methods

*Study descriptions*

The **Atherosclerosis Risk in Communities (ARIC)** study recruited 15,792 adults aged 45 to 64 years in 1987 through 1989 by probability sampling from Forsyth County, North Carolina; Jackson, Mississippi; suburbs of Minneapolis, Minnesota; and Washington County, Maryland.[[1](#_ENREF_1)] The Jackson sample comprised African Americans only; the other three samples represent the ethnic mix of their communities. Extensive information was collected at baseline on cardiovascular risk factors. The ARIC study was approved by the institutional review board of each field center institutes and participants gave informed consent including consent for genetic testing. In this study we included only European American participants.

Fibrinogen was measured at baseline in the entire ARIC cohort after an 8-hour fasting period. Circulating plasma fibrinogen was measured by the Clauss clotting rate method.[[2](#_ENREF_2)] Participants whose fibrinogen measurement was off 6SD from the mean were also excluded.

The **British 1958 birth cohort (B58C)** is a national population sample followed periodically from birth. At age 44-45 years, 9377 cohort members were examined by a research nurse in the home as described previously and non-fasting blood samples were collected with permission for DNA extraction and creation of immortalised cell cultures.[[3](#_ENREF_3)] DNA samples from unrelated subjects of white ethnicity, with nationwide geographic coverage, were genotyped either by the Wellcome Trust Case Control Consortium (WTCCC), the Type 1 Diabetes Genetics Consortium, or the GABRIEL consortium.[[4-6](#_ENREF_4)]

Details of the blood collection, fibrinogen measurement and covariate adjustment have been described elsewhere[[7](#_ENREF_7)]. In brief, fibrinogen was measured by the Clauss method using an MDA 180 coagulometer (Biomerieux, Basingstoke, UK) and adjusted for sex, laboratory batch, time of day, month of examination, and postal delay.[[2](#_ENREF_2)] Adjustment for age was not required as all subjects were aged 44-45 years. Use of anticoagulant therapy was a contraindication to blood sampling. Valid fibrinogen measurements were available for 6085 (93.7%) of the 6491 subjects with imputed genotypes.

The **Blue Mountains Eye Study (BMES)** is a population-based cohort study of eye diseases and other health outcomes in an urban population aged 49 years or older. In 1992-4, 3654 residents (82.4% of those eligible) aged 49+ years, living in two postcode areas near Sydney, Australia, participated; 2335 (75.1% of survivors) were re-examined after 5 years in 1997-9, and 1952 (76% of survivors) were re-examined after 10 years in 2002-4. The study was approved by the University of Sydney and the Sydney West Area Health Service Human Research Ethics Committees, and written, informed consent was obtained.[[8](#_ENREF_8),[9](#_ENREF_9)]

At baseline, eight-hour fasting blood samples were drawn from 3222 (88%) of 3654 participants for hematology and clinical biochemistry assessments. Blood samples were centrifuged into serum and plasma components and an aliquot of plasma sample was tested for fibrinogen within the same day as sample collection in the Institute of Clinical Pathology and Medical Research (ICPMR) at Westmead Hospital, west of Sydney. Plasma fibrinogen was measured by the Von Clauss assay using an ACL 300 coagulometer (IL-Coulter, Sydney), with a sensitivity of 50 mg/dL and an inter-assay CV of 10% at 200mg/dL.

The **Cardiovascular Health Study** **(CHS)** is a population-based cohort study of risk factors for CHD and stroke in adults ≥65 years conducted across 4 field centers.[[10](#_ENREF_10)] The original predominantly Caucasian cohort of 5,201 persons was recruited in 1989-1990 from random samples of the Medicare eligibility lists; subsequently, an additional predominantly African-American cohort of 687 persons was enrolled for a total sample of 5,888. DNA was extracted from blood samples drawn on all participants at their baseline examination in 1989-90. In this study we included only European American participants.

After an 8-12-h fast, CHS participants underwent phlebotomy by atraumatic venipuncture with a 21-gauge butterfly needle connected to a Vacutainer (Becton Dickinson, Rutherford, NJ) outlet via a Luer adaptor.[[11](#_ENREF_11)] For fibrinogen determination, an additional citrate-containing tube was processed at 4°C. The study measured fibrinogen levels using the Clauss methods.

The **Framingham Heart Study (FHS)** was started in 1948 with 5,209 randomly ascertained participants (the Original cohort) from Framingham, Massachusetts, US, who had undergone biannual examinations to investigate cardiovascular disease and its risk factors.[[12](#_ENREF_12)] In 1971, the Offspring cohort (comprising 5,124 children of the original cohort and the children's spouses) and in 2002, the Third Generation (consisting of 4,095 children of the Offspring cohort) were recruited. FHS participants in this study are of European ancestry. The methods of recruitment and data collection for the Offspring and Third Generation cohorts have been described.[[13](#_ENREF_13),[14](#_ENREF_14)]

Fibrinogen levels were measured using the Clauss method in the Offspring and the Third-generation subjects,[[2](#_ENREF_2)] and a modified method of Ratnoff and Menzie in the Original cohort subjects.[[15](#_ENREF_15)]

The **Gutenberg Health Study (GHS)** is designed as a population-based, prospective, observational single-center cohort study in the Rhein-Main-Region in western Mid-Germany to evaluate and improve cardiovascular risk stratification.[[16](#_ENREF_16)] The sample was drawn randomly from the governmental local registry offices in the city of Mainz and the district of Mainz-Bingen. The sample was stratified 1:1 for gender and for urban and rural residence with equal strata for decades of age. Individuals between 35 and 74 years of age were enrolled. In, total 15,010 study participants were included. A large variety of non-invasive cardiovascular phenotypes have been assessed and blood samples were drawn for biomarker measurements and genetic analyses. The study protocol and the sampling design were approved by the local ethics committee, and by the local and federal data safety commissioners. All participants gave written informed consent. Further, microarray-based analyses were performed in 3,891 individuals (SNP 6.0, Affymetrix, Santa Clara, CA). The analyses were subdivided into two batches, i.e. the microarray analyses of 1) 2,743 individuals were performed in 2008 (GHS I) of 1,148 individuals in 2009 (GHS II).

Fibrinogen was performed in citrated human plasma of 4,674 GHS participants. The analyses were done on the automated coagulation analyser BCS II (Siemens Diagnostics, Marburg, Germany) using reagents supplied by the manufacturer of the instrument. Assay plasma was brought to coagulation by a large excess of thrombin using the Multifibren U reagent (modification of the Clauss method).

The **GOYA** **(Male)** cohort is a longitudinal case-cohort (obese, non-obese) study comprising a randomly (1%) selected control group and all extremely overweight men identified among 362,200 Caucasian men examined at the mean age of 20 years at the draft boards in Copenhagen and its surrounding areas during 1943–1977. Obesity was defined as 35% overweight relative to a local standard in use at the time (mid 1970’s), corresponding to a BMI ≥31.0 kg/m2, which proved to be above the 99th percentile. All of the obese and 50% of the random sampled controls, who were still living in the region, were invited to a follow-up survey in 1992–94 at the mean age of 46 years, at which time the blood samples were taken and genotyping were performed for a total of 673 extremely overweight and 792 controls.[[17](#_ENREF_17)] With a sampling fraction of 0.5% (50% of 1%), the controls represent about 158,000 men among whom the case group was the most obese.

A functional photometric assay was employed to estimate fibrinogen concentration. The sample is mixed with a snake venom enzyme (Batroxobin) and fibrin formation is recorded turbidimetrically at 334 nm. Reaction conditions are such that a linear increase in absorbance is obtained over a concentration range of fibrinogen from 80-700 mg/dl. Higher or lower ranges can be measured by adjusting the sample volume. Calibration is performed with a single standard.[[18](#_ENREF_18)]

The **Hunter Community Study (HCS)** is a population-based prospective cohort study of community-dwelling men and women aged 55–85 years of age who reside in Newcastle, New South Wales (NSW), Australia. The cohort comprises 3253 participants that were randomly selected from the NSW State electoral roll between 2004 and 2007: details of recruitment have been published previously.[[19](#_ENREF_19)]

Citrated plasma was used for the fibrinogen assay, which was performed on a STAGO Evolution Expert Series platform using a Clauss technique. This was performed at baseline before the samples had been aliquoted for long term freezer storage.

The **Aging in the Chianti Area (InCHIANTI)** study is a population-based epidemiological study aimed at evaluating the factors that influence mobility in the older population living in the Chianti region in Tuscany, Italy. The details of the study have been previously reported.[[20](#_ENREF_20)] Briefly, 1616 residents were selected from the population registry of Greve in Chianti (a rural area: 11,709 residents with 19.3% of the population greater than 65 years of age), and Bagno a Ripoli (Antella village near Florence; 4,704 inhabitants, with 20.3% greater than 65 years of age). The participation rate was 90% (n=1453).

Overnight fasted blood samples were used for genomic DNA extraction, and measurement of fibrinogen. Plasma fibrinogen concentrations were measured by the Clauss method using STA fibrinogen assay (Diagnostic Stago, Roche Diagnostics, France)[[2](#_ENREF_2)]. The intra- and inter-assay CV was 4.1%.

The **Lothian Birth Cohort (LBC) studies**, **LBC1936 & LBC1921,** were ascertained as follows.

The LBC1936 consists of 1,091 relatively healthy individuals assessed on cognitive and medical traits at 70 years of age. They were born in 1936, most took part in the Scottish Mental Survey of 1947, and almost all lived independently in the Lothian region of Scotland (Edinburgh City and surrounding area). A full description of participant recruitment and testing can be found elsewhere.[[21](#_ENREF_21),[22](#_ENREF_22)] The LBC1921 cohort consists of 550 relatively healthy individuals, 316 females and 234 males, assessed on cognitive and medical traits at 79 years of age. They were born in 1921, most took part in the Scottish Mental Survey of 1932, and almost all lived independently in the Lothian region in Scotland. A full description of participant recruitment and testing can be found elsewhere.[[21](#_ENREF_21),[23](#_ENREF_23)] Ethics permission for the study was obtained from the Multi-Centre Research Ethics Committee for Scotland (MREC/01/0/56) and from Lothian Research Ethics Committee (LBC1936: LREC/2003/2/29 and LBC1921: LREC/1998/4/183). The research was carried out in compliance with the Helsinki Declaration. All subjects gave written, informed consent.

Fibrinogen levels were measured using HemosIL^TM^ based on the Clauss method. No exclusions were applied. Outliers were removed (>3.3SD). Plasma fibrinogen was in g/L, natural log transformed.

The **Ludwigshafen Risk and Cardiovascular Health (LURIC)** study is an ongoing prospective study of more than 3,300 individuals of German ancestry in whom cardiovascular and metabolic phenotypes (CAD, MI, dyslipidemia, hypertension, metabolic syndrome and diabetes mellitus) have been defined or ruled out using standardized methodologies in all study participants. Inclusion criteria for LURIC were: German ancestry (limitation of genetic heterogeneity), clinical stability (except for acute coronary syndromes) and availability of a coronary angiogram. Exclusion criteria were: any acute illness other than acute coronary syndromes, any chronic disease where non-cardiac disease predominated and a history of malignancy within the last five years. Genome-wide analyses using the Affymetrix 6.0 have been completed in all participants. A 10-year clinical follow-up for total and cause specific mortality has been completed.

Fasting blood samples were collected at baseline in the morning before angiography. Fibrinogen was measured in citrate plasma using the Clauss method (STA fibrinogen/STA Stago, Stago Diagnostica/Roche Mannheim, Germany) at the Haemostaseology Laboratory of the Ludwigshafen hospital on a daily basis.

As part of a **Netherlands Twin Registry (NTR)** biobank project, 9,530 participants from 3,477 families were visited at home between January 2004 and July 2008 for collection of blood samples. Visits were scheduled between 7:00 and 10:00 am and fertile women were bled on day 2–4 of the menstrual cycle, or in their pill-free week. Fertile women were bled on day 2–4 of the menstrual cycle, or in their pill-free week. Body composition was measured and information about physical health and lifestyle (e.g. smoking and drinking behavior, physical exercise, medication use) was obtained. For more detailed information about the methodology of the NTR Biobank study, see.[[24](#_ENREF_24)] The NTR studies were approved by the Central Ethics Committee on Research involving human subjects of the VU University Medical Center, Amsterdam, an Institutional Review Board certified by the US Office of Human Research Protections (IRB number IRB-2991 under Federal wide Assurance-3703; IRB/institute codes, NTR 03-180). All subjects provided written informed consent. Valid GWA data were available for 6171 individuals.

Fibrinogen was measured in a 4.5 ml CTAD tube that was stored during transport in melting ice and upon arrival at the laboratory, centrifuged for 20 minutes at 2000x g at 4° C, after which citrated plasma was harvested, aliquoted (0.5 ml), snapfrozen in dry ice, and stored at –30° C. Fibrinogen levels were determined on a STA Compact Analyzer (Diagnostica Stago, France), using STA Fibrinogen (Diagnostica Stago, France).

The **Precocious Coronary Artery Disease Study (PROCARDIS)** consists of coronary artery disease (CAD) cases and controls from four European countries (UK, Italy, Sweden and Germany). CAD (defined as myocardial infarction, acute coronary syndrome, unstable or stable angina, or need for coronary artery bypass surgery or percutaneous coronary intervention) was diagnosed before 66 years of age and 80% of cases had a sibling fulfilling the same criteria for CAD. Subjects with self-reported non-European ancestry were excluded. Among the “genetically-enriched” CAD cases, 70% had suffered myocardial infarction (MI).

Plasma fibrinogen concentrations were measured in fasting citrate plasma samples by the Clauss method using the IL Test Fibrinogen C kit and IL Test Calibration Plasma, on the ACL-9000 coagulometer (all from Instrumentation Laboratory Spa, Milan, Italy).[[2](#_ENREF_2)] The inter-assay CV was 7% (n=106).

**PROspective Study of Pravastatin in the Elderly at Risk (PROSPER)** was a prospective multicenter randomized placebo-controlled trial to assess whether treatment with pravastatin diminishes the risk of major vascular events in elderly. Between December 1997 and May 1999, we screened and enrolled subjects in Scotland (Glasgow), Ireland (Cork), and the Netherlands (Leiden). Men and women aged 70-82 years were recruited if they had pre-existing vascular disease or increased risk of such disease because of smoking, hypertension, or diabetes. A total number of 5804 subjects were randomly assigned to pravastatin or placebo. A large number of prospective tests were performed including Biobank tests and cognitive function measurements. A detailed description of the study has been published elsewhere.[[25](#_ENREF_25),[26](#_ENREF_26)]

Fibrinogen levels were measured by the Clauss method using aMDA180 coagulometer (Trinity Biotech; calibrant 9th British standard National Institute for Biological Standards and Control).[[2](#_ENREF_2)]

The **Rotterdam Study (RS-I and RS-II)** is a prospective, population-based cohort study of determinants of several chronic diseases in older adults.[[27](#_ENREF_27)] RS-I comprised 7,983 inhabitants of Ommoord, a district of Rotterdam in the Netherlands, who were 55 years or over. The baseline examination took place between 1990 and 1993. In 1999, the cohort was extended to include 3011 inhabitants who reached the age of 55 years after the baseline examination and persons aged 55 years or older who migrated into the research area (RS-II). Subjects are of European ancestry based on their self-report.

In RS-I, fibrinogen levels were derived at baseline (RS-I-1) from the clotting curve of the prothrombin time assay using Thromborel S as a reagent on an automated coagulation laboratory 300 (ACL 300, Instrumentation Laboratory, Zaventem, Belgium). At the second follow up of RS-I (RS-I-3) and the baseline visit of RS-II, fibrinogen levels were derived from the clotting curve of the prothrombin time assay using Thromborel S (Behringwerke, Marburg, Germany) as a reagent on an automated coagulation analyzer (Sysmex CA-500 Series Systems, Siemens, Breda, the Netherlands).

The **SardiNIA study** has been previously described.[[28](#_ENREF_28)] Briefly, it is a large population-based study which consists of 6,921 individuals, males and females, ages 14-102 y, and representing >60% of the adult population of four villages in the Lanusei Valley of Sardinia. Samples have been characterized for several quantitative traits and medical conditions, including fibrinogen.

Fibrinogen levels were measured using the Clauss method.[[2](#_ENREF_2)]

The **Study of Health in Pomerania (SHIP)** is a cohort study in West Pomerania, the north-east area of Germany and has been described previously.[[29](#_ENREF_29),[30](#_ENREF_30)] From the entire study population of 212,157 inhabitants living in the area, a sample was selected from the population registration offices, where all German inhabitants are registered. Only individuals with German citizenship and main residency in the study area were included. A two-stage cluster sampling method was adopted from the WHO MONICA Project Augsburg, Germany. In a first step, the three cities of the region (with 17,076 to 65,977 inhabitants) and the 12 towns (with 1,516 to 3,044 inhabitants) were selected. Further 17 out of 97 smaller towns (with less than 1,500 inhabitants) were drawn at random. In a second step, from each of the selected communities, subjects were drawn at random, proportional to the population size of each community and stratified by age and gender. Finally, 7,008 subjects aged 20 to 79 years were sampled, with 292 persons of each gender in each of the twelve five-year age strata. In order to minimize drop-outs by migration or death, subjects were selected in two waves. The net sample (without migrated or deceased persons) comprised 6,267 eligible subjects. The SHIP population finally comprised 4,308 participants at baseline (corresponding to a final response of 68.8%).

A non-fasting blood sample was drawn from the antecubital vein in the supine position and immediately analyzed or stored at -80°C. Plasma fibrinogen concentrations were assayed according to Clauss using an Electra 1600 analyzer (Instrumentation Laboratory, Barcelona, Spain).[[2](#_ENREF_2)] Coagulation time is measured and transferred into the result in g/L by applying a reference curve calculated in the laboratory. The assay proves linearity between 0.7 – 7 g/L. The analytical sensitivity of the assay was 0.7 g/L. Internal quality control measures were performed daily using two levels of manufacturers’ control materials. External quality control measures were performed on a regular basis by participating in analysis programs. The inter-assay coefficients of variation were 4.61 % at low levels (mean value = 0.95 g/L) and 1.82% at high levels (mean value = 3.22 g/L) of control material.

The **TwinsUK** cohort was derived from the UK adult twin registry based at King’s College London (www.twinsUK.ac.uk). These unselected twins have been recruited from the general population through national media campaigns in the United Kingdom and shown to be comparable to age-matched population singletons in terms of disease-related and lifestyle characteristics.[[31](#_ENREF_31)] Informed consent was obtained from all participants and the study was approved by the St. Thomas' Hospital Ethics Committee.

Fasting blood samples was taken from samples into 0.13 trisodium citrate containers (Becton Dickinson, Oxford, United Kingdom) at room temperature, centrifuged at 2560*g* for 20 minutes to obtain platelet-poor plasma within 1 hour of collection and stored at –40°C until analysis. Fibrinogen levels were determined using the Clauss method.[[2](#_ENREF_2),[32](#_ENREF_32)]

The **Women’s Genome Health Study (WGHS)** is a prospective cohort of initially healthy, female North American health care professionals at least 45 years old at baseline representing participants in the Women’s Health Study (WHS) who provided a blood sample at baseline and consent for blood-based analyses.[[33](#_ENREF_33)] The WHS was a 2x2 trial beginning in 1992-1994 of vitamin E and low dose aspirin in prevention of cancer and cardiovascular disease with about 10 years of follow-up. Since the end of the trial, follow-up has continued in observational mode. Additional information related to health and lifestyle were collected by questionnaire throughout the WHS trial and continuing observational follow-up.

Fibrinogen in plasma from the baseline blood sample was measured by a mass-based immunoturbidimetric assay (DiaSorin) with reproducibility of 5.20% and 3.99% at concentrations of 0.99 and 2.74 g/L respectively.

**References**

1. ARIC (1989) The Atherosclerosis Risk in Communities (ARIC) Study: design and objectives. The ARIC investigators. Am J Epidemiol 129: 687-702.

2. Clauss A (1957) [Rapid physiological coagulation method in determination of fibrinogen]

Gerinnungsphysiologische Schnellmethode zur Bestimmung des Fibrinogens. Acta Haematol 17: 237-246.

3. Strachan DP, Rudnicka AR, Power C, Shepherd P, Fuller E, et al. (2007) Lifecourse influences on health among British adults: effects of region of residence in childhood and adulthood. Int J Epidemiol 36: 522-531.

4. Barrett JC, Clayton DG, Concannon P, Akolkar B, Cooper JD, et al. (2009) Genome-wide association study and meta-analysis find that over 40 loci affect risk of type 1 diabetes. Nat Genet 41: 703-707.

5. Moffatt MF, Gut IG, Demenais F, Strachan DP, Bouzigon E, et al. (2010) A large-scale, consortium-based genomewide association study of asthma. N Engl J Med 363: 1211-1221.

6. WTCCC (2007) Genome-wide association study of 14,000 cases of seven common diseases and 3,000 shared controls. Nature 447: 661-678.

7. Rudnicka AR, Rumley A, Lowe GD, Strachan DP (2007) Diurnal, seasonal, and blood-processing patterns in levels of circulating fibrinogen, fibrin D-dimer, C-reactive protein, tissue plasminogen activator, and von Willebrand factor in a 45-year-old population. Circulation 115: 996-1003.

8. Attebo K, Mitchell P, Smith W (1996) Visual acuity and the causes of visual loss in Australia. The Blue Mountains Eye Study. Ophthalmology 103: 357-364.

9. Mitchell P, Smith W, Attebo K, Wang JJ (1995) Prevalence of age-related maculopathy in Australia. The Blue Mountains Eye Study. Ophthalmology 102: 1450-1460.

10. Fried LP, Borhani NO, Enright P, Furberg CD, Gardin JM, et al. (1991) The Cardiovascular Health Study: design and rationale. Ann Epidemiol 1: 263-276.

11. Cushman M, Cornell ES, Howard PR, Bovill EG, Tracy RP (1995) Laboratory methods and quality assurance in the Cardiovascular Health Study. Clin Chem 41: 264-270.

12. Dawber TR, Kannel WB, Lyell LP (1963) An approach to longitudinal studies in a community: the Framingham Study. Ann N Y Acad Sci 107: 539-556.

13. Splansky GL, Corey D, Yang Q, Atwood LD, Cupples LA, et al. (2007) The Third Generation Cohort of the National Heart, Lung, and Blood Institute's Framingham Heart Study: design, recruitment, and initial examination. Am J Epidemiol 165: 1328-1335.

14. Feinleib M, Kannel WB, Garrison RJ, McNamara PM, Castelli WP (1975) The Framingham Offspring Study. Design and preliminary data. Prev Med 4: 518-525.

15. Kannel WB, Wolf PA, Castelli WP, D'Agostino RB (1987) Fibrinogen and risk of cardiovascular disease. The Framingham Study. JAMA 258: 1183-1186.

16. Wild PS, Zeller T, Beutel M, Blettner M, Dugi KA, et al. (2012) [The Gutenberg Health Study]

Die Gutenberg Gesundheitsstudie. Bundesgesundheitsblatt Gesundheitsforschung Gesundheitsschutz 55: 824-829.

17. Paternoster L, Evans DM, Nohr EA, Holst C, Gaborieau V, et al. (2011) Genome-wide population-based association study of extremely overweight young adults--the GOYA study. PLoS One 6: e24303.

18. Becker U, Bartl K, Wahlefeld AW (1984) A functional photometric assay for plasma fibrinogen. Thromb Res 35: 475-484.

19. McEvoy M, Smith W, D'Este C, Duke J, Peel R, et al. (2010) Cohort profile: The Hunter Community Study. Int J Epidemiol 39: 1452-1463.

20. Ferrucci L, Bandinelli S, Benvenuti E, Di Iorio A, Macchi C, et al. (2000) Subsystems contributing to the decline in ability to walk: bridging the gap between epidemiology and geriatric practice in the InCHIANTI study. J Am Geriatr Soc 48: 1618-1625.

21. Deary IJ, Gow AJ, Pattie A, Starr JM (2012) Cohort profile: the Lothian Birth Cohorts of 1921 and 1936. Int J Epidemiol 41: 1576-1584.

22. Deary IJ, Gow AJ, Taylor MD, Corley J, Brett C, et al. (2007) The Lothian Birth Cohort 1936: a study to examine influences on cognitive ageing from age 11 to age 70 and beyond. BMC Geriatr 7: 28.

23. Deary IJ, Whiteman MC, Starr JM, Whalley LJ, Fox HC (2004) The impact of childhood intelligence on later life: following up the Scottish mental surveys of 1932 and 1947. J Pers Soc Psychol 86: 130-147.

24. Willemsen G, de Geus EJ, Bartels M, van Beijsterveldt CE, Brooks AI, et al. (2010) The Netherlands Twin Register biobank: a resource for genetic epidemiological studies. Twin Res Hum Genet 13: 231-245.

25. Shepherd J, Blauw GJ, Murphy MB, Bollen EL, Buckley BM, et al. (2002) Pravastatin in elderly individuals at risk of vascular disease (PROSPER): a randomised controlled trial. Lancet 360: 1623-1630.

26. Shepherd J, Blauw GJ, Murphy MB, Cobbe SM, Bollen EL, et al. (1999) The design of a prospective study of Pravastatin in the Elderly at Risk (PROSPER). PROSPER Study Group. PROspective Study of Pravastatin in the Elderly at Risk. Am J Cardiol 84: 1192-1197.

27. Global Lipids Genetics Consortium, Willer CJ, Schmidt EM, Sengupta S, Peloso GM, et al. (2013) Discovery and refinement of loci associated with lipid levels. Nat Genet 45: 1274-1283.

28. Pilia G, Chen WM, Scuteri A, Orru M, Albai G, et al. (2006) Heritability of cardiovascular and personality traits in 6,148 Sardinians. PLoS Genet 2: e132.

29. John U, Greiner B, Hensel E, Ludemann J, Piek M, et al. (2001) Study of Health In Pomerania (SHIP): a health examination survey in an east German region: objectives and design. Soz Praventivmed 46: 186-194.

30. Volzke H, Alte D, Schmidt CO, Radke D, Lorbeer R, et al. (2011) Cohort profile: the study of health in Pomerania. Int J Epidemiol 40: 294-307.

31. Andrew T, Hart DJ, Snieder H, de Lange M, Spector TD, et al. (2001) Are twins and singletons comparable? A study of disease-related and lifestyle characteristics in adult women. Twin Res 4: 464-477.

32. Dunn EJ, Ariens RA, de Lange M, Snieder H, Turney JH, et al. (2004) Genetics of fibrin clot structure: a twin study. Blood 103: 1735-1740.

33. Ridker PM, Chasman DI, Zee RY, Parker A, Rose L, et al. (2008) Rationale, design, and methodology of the Women's Genome Health Study: a genome-wide association study of more than 25,000 initially healthy american women. Clin Chem 54: 249-255.
